# 3D meshwork architecture of the outer coat protein CotE: implications for bacterial endospore sporulation and germination

**DOI:** 10.1128/mbio.02472-24

**Published:** 2025-03-06

**Authors:** Dukwon Lee, Yeongjin Baek, Migak Park, Doyeon Kim, Kyumi Byun, Jaekyung Hyun, Nam-Chul Ha

**Affiliations:** 1 Department of Agricultural Biotechnology, Research Institute of Agriculture and Life Sciences, Center for Food and Bioconvergence, Interdisciplinary Programs in Agricultural Genomics, CALS, Seoul National University, Seoul, South Korea; 2 School of Biological Sciences, Institute of Molecular Biology and Genetics, Seoul National University, Seoul, South Korea; 3 School of Pharmacy, Sungkyunkwan University, Suwon, South Korea; University of Pittsburgh, Pittsburgh, Pennsylvania, USA; New York University, New York, New York, USA

**Keywords:** CotE, endospore, *Bacillus cereus*, cryo-electron microscopy, cryo-electron tomography

## Abstract

*Bacillus cereus,* a Gram-positive aerobic bacterium commonly found in soil, food, and water, forms endospores that can withstand harsh environmental conditions. The endospores are encased in a protective spore coat consisting of multiple layers of proteins, among which, CotE serves as a crucial morphogenetic protein within the outer coat. In this study, we observed that the homotrimeric CotE protein underwent further oligomerization induced by Ca^2+^ and was subsequently dissociated by dipicolinic acid, a compound released from the spore core during germination. Through cryo-electron microscopy and tomography analyses of the Ca^2+^-induced CotE oligomer, combined with structural predictions and biochemical studies, we propose a three-dimensional meshwork organization facilitated by tryptophan-based interactions between CotE trimers. The resulting meshwork was organized in a defective diamond-like tetrahedral configuration. These insights enhance our understanding of how CotE contributes to endospore morphogenesis and germination through the rapid disassembly of these layers.

**IMPORTANCE:**

Bacterial endospores are highly resilient structures that allow bacteria to survive extreme environmental conditions, making them a significant concern in food safety and healthcare. The protein CotE plays a critical role in forming the protective outer coat of these endospores. Our research uncovers the three-dimensional meshwork architecture of CotE and reveals how it contributes to the structural integrity and rapid disassembly of endospores during germination. By understanding CotE’s unique 3D structure and its interaction with other molecules, we gain valuable insights into how bacterial endospores are formed and how they can be effectively targeted for sterilization. This work not only advances our fundamental knowledge of bacterial endospore biology but also has potential applications in developing new strategies to combat bacterial contamination and improve sterilization techniques in the food and healthcare industries.

## INTRODUCTION

The endospore, a survival mechanism in certain Gram-positive bacteria like *Bacillus* and *Clostridium* species, forms in response to extreme environmental conditions such as nutrient deprivation or sudden oxygen fluctuations, exhibiting remarkable resilience under these challenges (1–4). Despite this resilience, endospores can germinate rapidly and revert to vegetative growth under favorable conditions (5). However, the intricate molecular mechanisms governing both sporulation and germination remain to be elucidated. Notably, the thermal resistance exhibited by spores of several species of *Clostridium* and *Bacillus* poses a formidable challenge to food safety and preservation (6).

*Bacillus* endospores are divided into several layers with different functions: the core, cortex, outer and inner coats, and crust or exosporium (7). The outer coat is thought to play a crucial role in potentiating the environmental resistance of endospores (8). CotE is a key morphogenetic protein of the outer coat (7, 9, 10), with a molecular weight of approximately 20 kDa. The deletion of *cotE* led to the disappearance of not only the outer coat but also all structures outside the outer coat layer in *Bacillus subtilis* (8, 9, 11).

The structure of CotE has only been studied at a low resolution. In 1992, the filamentous structure of refolded and purified *B. subtilis* CotE was observed using negative stain electron microscopy (EM) (12). In 2015, a honeycomb-like meshwork of *B. subtilis* CotE protein, expressed in *E. coli,* was observed (13). Recently, a bead-like layer was observed exclusively in the presence of CotE during mid-engulfment in sporulating *B. subtilis* cells using cryo-electron tomography (cryo-ET) (11).

Dipicolinic acid (DPA) is of significant interest in biology and chemistry because of its distinctive and critical role in endospores (3). DPA fills the spore core region, comprising 5%–15% of the endospore dry weight (14). Its capacity to chelate divalent metal ions, especially Ca^2+^, to form a stable and low-solubility complex known as calcium dipicolinate (CaDPA), is well documented (15). The deposition of CaDPA endows bacterial spores with extraordinary durability in the core region that contains the DNA (16).

Moreover, DPA acts as a signaling molecule during spore germination, triggering the release and activation of enzymes for spore germination and bacterial proliferation (17). CaDPA is released from the spore core into the outer layers of endospores via the channel protein SpoVA, resulting in endospore disintegration (18). In a recent study, the structure of an amino acid-gated K^+^ channel (GerA complex) expressed during the germination stage was predicted by the AlphaFold2, and extensive functional studies were performed (19).

In this study, we found that Ca^2+^ and DPA influenced the physicochemical properties of purified CotE. Cryo-electron microscopy (cryo-EM) and cryo-ET analyses, combined with structural predictions, demonstrated the molecular architecture of the CotE meshwork. Furthermore, biochemical and mutational analyses accounted for the molecular basis of CotE-mediated encasement and germination.

## RESULTS

### Ca^2+^ and DPA alter the oligomeric states of the CotE protein

**Fig 1:**
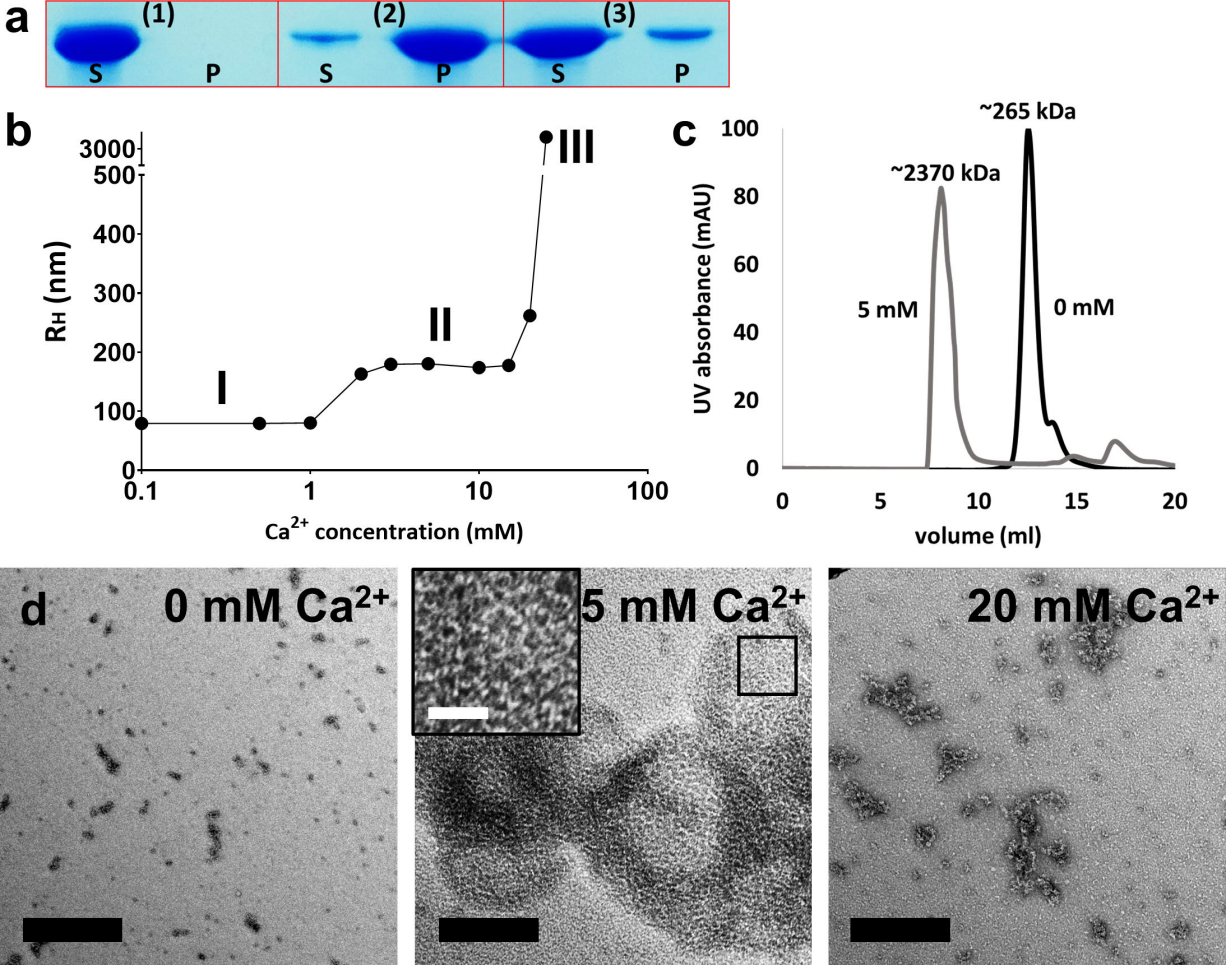
Initial characterization of the purified *B. cereus* CotE protein. (**a**) Ca^2+^-induced precipitation and DPA-mediated resolubilization of CotE (1). The CotE protein was initially solubilized in a buffer (20 mM tris, pH 8.0) (2). CotE was precipitated by the addition of 50 mM CaCl_2_, and (3) resolubilized CotE was obtained from the precipitate formed in (2) by adding 5 mM DPA. fractions labeled “S” (supernatant) and “P” (precipitate) were collected post-centrifugation at 20,000 × *g* for 10 min to discern the solubility status of CotE under each condition. (**b**) Changes in the hydrodynamic radius (R_H_) of the CotE protein in response to Ca^2+^, as measured using DLS. The plot was segmented into three distinct phases based on the behavior of the R_H_ as the Ca^2+^ concentration increased: I, II, and III. (**c**) SEC analysis of CotE at two CaCl_2_ concentrations (0 and 5 mM). Two chromatograms are shown: the CotE protein (5 mg/mL) in SEC buffer without CaCl₂ is depicted by a black line, whereas the CotE protein in the presence of 5 mM CaCl₂ is represented by a gray line. Peaks corresponding to the different forms of CotE are annotated with their estimated molecular weights, calculated using the protein size reference shown in Fig. S20. (**d**) Negative-stain electron microscopy images of purified CotE at various Ca^2+^ concentrations. The structural morphology of the purified CotE protein in the SEC buffer is displayed at different Ca^2+^ concentrations: 0 mM (left), 5 mM (middle), and 20 mM (right). Black scale bars represent 200 nm. The inset in the middle image presents an enlarged view of the boxed region, showing mesh-like structures with an approximate diameter of 20 nm forming a honeycomb-like network. The white scale bar represents 30 nm.

During the purification of CotE from *Bacillus cereus*, we observed that the recombinant CotE protein formed aggregates in the presence of Ca^2+^. Similar aggregation was induced by Mn^2+^ and Ni^2+^ at a concentration of 5 mM, but was only partially induced by Mg^2+^ (Fig. S1). However, no aggregation was observed with divalent ions at a concentration of 0.1 mM. This study focused solely on the role of Ca^2+^ due to its physiological relevance (see Discussion for details). We further found that the Ca^2+^-induced protein aggregate was resolubilized by DPA (Fig. 7b through d), a well-known Ca^2+^-chelating agent involved in the germination process (20). The CotE protein was purified without an affinity chromatography step through a process involving Ca^2+^-induced aggregation and DPA-dependent resolubilization, followed by further purification using size-exclusion chromatography (SEC). The resulting CotE protein was used for subsequent biochemical and structural analyses (Fig. S2; Fig. 1a).

We investigated the CotE protein in the presence of Ca^2+^ using dynamic light scattering (DLS) to measure its molecular size in solution. The CotE-containing solution exhibited three distinct phases in terms of the average hydrodynamic radius (R_H_) as the Ca^2+^ concentration increased in the buffer (Fig. 1b). CotE showed an R_H_ value <100 nm for Ca^2+^ concentrations <1 mM (Phase I), and the R_H_ of CotE increased twofold between 1 and 20 mM Ca^2+^ (Phase II). Above 20 mM Ca^2+^, CotE formed large species with extremely high R_H_ (Phase III).

We further analyzed the molecular size of CotE in the absence and presence of Ca^2+^ using SEC. The CotE protein exhibited an apparent molecular weight corresponding to a 12-mer in Ca-free buffer and a larger oligomer (>100 mer) in 5 mM Ca^2+^-containing buffer in SEC (Fig. 1c). CotE formed a visible precipitate above 20 mM Ca^2+^, which could not be analyzed by SEC. Therefore, our findings indicate that higher Ca^2+^ levels are responsible for the higher oligomerization of CotE.

### Honeycomb-like meshwork pattern of CotE in the presence of 5 mM Ca^2+^ in negative-stain EM

We conducted negative-stain EM to investigate the CotE protein by varying the Ca^2+^ concentrations (0, 5, and 20 mM). In the absence of Ca^2+^, CotE molecules appeared as small particles without any characteristic aggregation (Fig. 1d, left). Upon exposure to 5 mM Ca^2+^, the proteins exhibited extensive network formation resembling a honeycomb pattern (Fig. 1d, middle). Notably, this pattern closely resembles the shape previously observed for the *B. subtilis* CotE protein (13). At 20 mM Ca^2+^, only larger aggregates without any regular patterns were observed in the EM images (Fig. 1d, right). Our findings highlighted the role of Ca^2+^ in triggering the oligomerization of CotE proteins, emphasizing the formation of mesh-like structures at 5 mM concentration, which can be further analyzed using cryo-EM.

### Cryo-EM structure of the Ca-CotE meshwork at a pseudoatomic resolution

**Fig 2:**
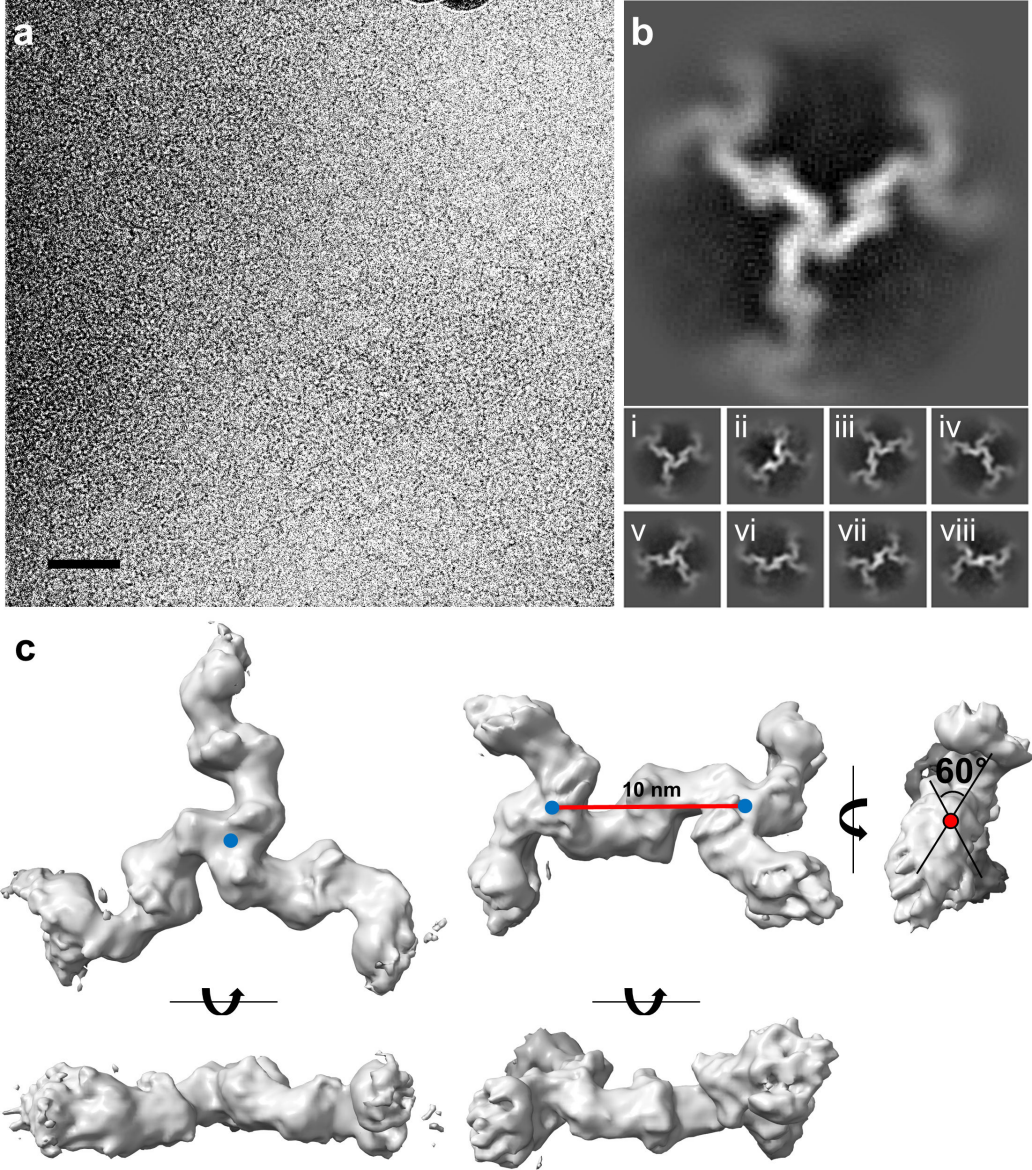
Cryo-EM analysis of the CotE meshwork at 5 mM CaCl_2_. (**a**) A representative cryo-EM image of the CotE meshwork formed in the presence of 5 mM CaCl_2_. The scale bar indicates 50 nm. (**b**) 2D classification images of the CotE protein structure. The representative 2D class is shown in the top panel, with eight additional classes depicted in the bottom panel (i–viii). (**c**) The two 3D models were derived from 3D classification. The C3 symmetry model is shown on the left, and the C2 symmetry model is shown on the right. The two models are displayed in orthogonal views. The 60° torsion angle along the inter-trimer axis (red line) connecting the two trimeric centers (blue circles, distance of 10 nm) is illustrated in the right model. The resolution graphs for gold-standard Fourier shell correlation (GSFSC) are presented in Fig. S21. Data collection and processing statistics are presented in Table S2.

To reveal the structural features of the Ca-bound CotE protein (Ca-CotE) in solution, distinguished by its net-like formation, we performed cryo-EM on the Ca-CotE sample in the presence of 5 mM Ca^2+^. Cryo-EM imaging revealed a polygonal net-like pattern (Fig. 2a). Through a 2D classification analysis of this pattern, we identified two principal connections within the central region of the images, centered at the Y-shaped trimer units (Fig. 2b, classes i, ii, iii, iv, and v) and the staggered connection between the trimers (classes vi, vii, and viii). By overlapping these two connections, it is possible to interconnect them, thereby constructing a larger singular structural entity. Notably, the diffused outskirts in both 2D averaged images suggest a degree of flexibility in the assembled structure.

The 3D reconstructions of the CotE cryo-EM electron map yielded two distinct 3D models: a radiating Y-shaped trimeric unit (C3 symmetry model at 6.55 Å, Fig. 2c left) and a joined assembly of two Y-shaped trimers (C2 symmetry model at 6.86 Å, Fig. 2c right). The construction flowchart and orientation plot for each EM map are shown in Fig. S3 and S4. The C2 symmetry model revealed a distance of 10 nm between the two Y-shaped trimers and a notable twist, with a torsion angle of approximately 60° along the axis connecting their centers (see Fig. 2c, right). The two 3D models represented different parts of a single CotE meshwork arrangement. As these CotE meshworks are formed in the presence of Ca^2+^, our findings suggest that the oligomerization of CotE induced by Ca^2+^ is governed by specific molecular interactions rather than nonspecific or random interactions between CotE molecules.

### Matched CotE structures predicted by AlphaFold2

**Fig 3:**
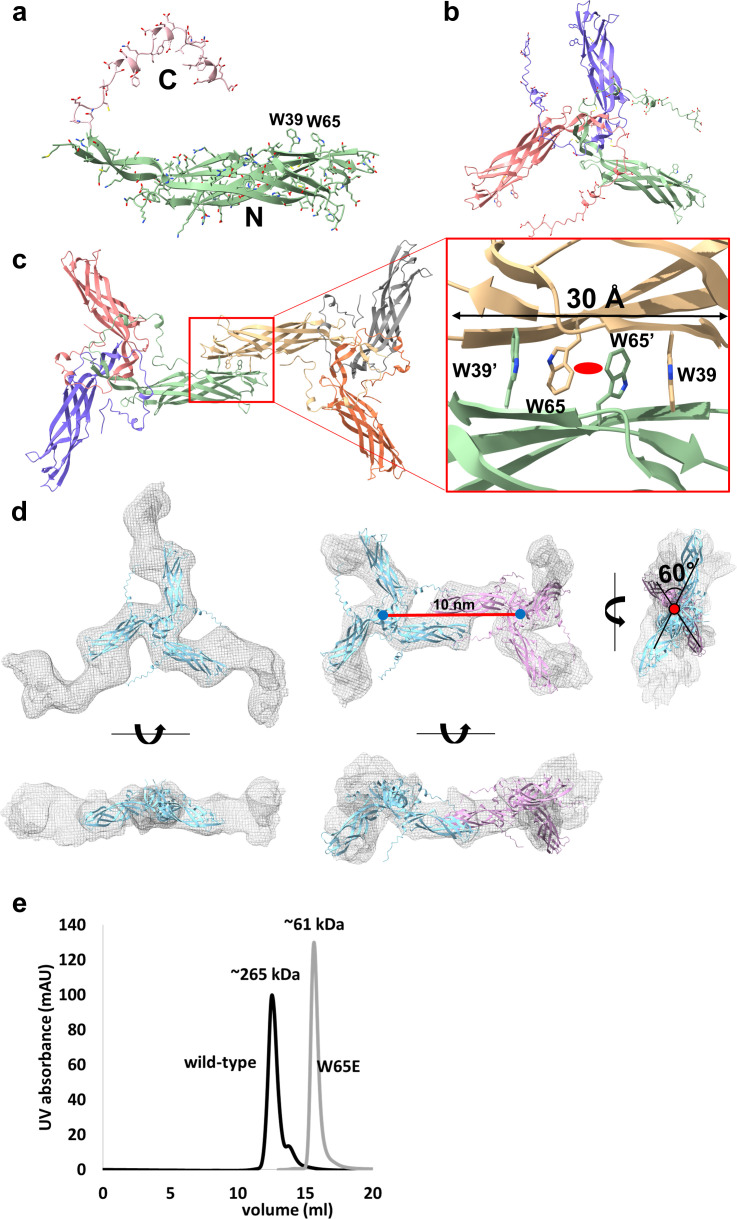
AlphaFold2-predicted CotE models and their superposition in cryo-EM maps reveal Trp65 as the center of dimeric interaction. (**a**) The CotE monomer model from the AlphaFold database (AF-A0A158RFX0-F1). The N-terminal body domain (N) is shown in green, and the C-terminal tail region (C) is shown in pink. Trp39 and Trp65 are labeled. (**b**) The trimeric model of CotE generated by AlphaFold2. The PAE and pLDDT plots for the model are shown in Fig. S5. Each subunit is distinctly colored for clarity: red, green, and violet. The hydrophobic core at the center of the trimer is enlarged in Fig. S6. (**c**) The hexameric model of CotE generated by AlphaFold2 serves as a speculative framework to explore potential structural arrangements. The PAE and pLDDT plots for the model are shown in Fig. S9, highlighting significant predicted errors (>20 Å) in the trimer-trimer distances. The two trimers are hypothesized to be joined by π–π stacking interactions of Trp39 (W39 and W39′) and Trp65 (W65 and W65′) at the molecular twofold axis (red oval), which is enlarged in the right panel. The length of the interaction patch between the trimers measures 30 Å. (**d**) Structural superposition of the AlphaFold2-predicted trimer models onto the two cryo-EM maps shown in Fig. 2c. The superposition was performed using ChimeraX (22) and refined with Phenix (23). Each trimer model is represented in cartoon format, with one colored light blue and the other pink. An enlarged view of the dimeric interface between the two trimers is presented in Fig. S10. (**e**) Comparative SEC analysis of the W65E-mutant CotE protein versus the wild-type protein. The estimated molecular weights are marked above each peak. Note that the SEC profile for the wild-type protein is reproduced from Fig. 1c. The SEC multi-angle light scattering (MALS) result for the W65E mutant CotE protein (70 kDa) is presented in Fig. S12, corroborating the findings of this SEC analysis.

The limited resolution of the cryo-EM map of the CotE meshwork precludes atomic-level structure elucidation. AlphaFold2 was used to generate predicted structures for interpreting the low-resolution cryo-EM map (21). The CotE monomer in the AlphaFold Protein Structure Database (https://alphafold.ebi.ac.uk) predominantly consists of an N-terminal domain (residues 1–146) and an unstructured C-terminal tail (residues 147–180), as depicted in Fig. 3a.

AlphaFold2 (version 2.3) (21) predicted the Y-shaped trimeric structures with high confidence, as supported by the pLDDT and PAE scores (Fig. S5). The models showed a compact hydrophobic core within the trimer N-terminal domain and unstructured C-terminal tails extending outward (Fig. 3b; Fig. S6). Notably, the evolutionarily conserved Trp39 and Trp65 residues (Fig. S7) were prominently displayed on the surface of the distal end of the N-terminal domain (Fig. 3a), and the unstructured C-terminal tail contained 15 negatively charged residues (Fig. S8). More importantly, AlphaFold2 (21) predicted a hexameric assembly of two Y-shaped trimers (Fig. 3c; Fig. S9). The trimers engage in π-π interactions through a 30 Å binding interface between Trp39 and Trp65 at the twofold symmetry axis, with Trp65 residues positioned centrally and Trp39 residues on the periphery (Fig. 3c).

The AlphaFold2-predicted models aligned well with the low-resolution features of the cryo-EM maps, particularly capturing the N-terminal domains of the Y-shaped trimers (Fig. 3d). Despite the inability of the cryo-EM map to resolve the C-terminal tails, the predicted N-terminal domains of the Y-shaped trimers reasonably consistent with the cryo-EM data. The hexameric cryo-EM model was constructed by fitting two AlphaFold2 trimer models to the C2-symmetry cryo-EM map, followed by energy minimization (Fig. 3d; Fig. S10). Dimeric interactions mediated by conserved tryptophan residues are depicted in the cryo-EM map. The 10 nm distance between the two Y-shaped bodies shown in Fig. 2c indicates the distance between the two CotE trimer cores. A notable deviation was observed in the torsion angles along the axis connecting the trimer centers (~20° in the predicted model and ~60° in the model fitted to the cryo-EM map) (Fig. 3d; Fig. S11). These observations indicate that Ca²^+^ modulates the function of the C-terminal tails, as supported by the reduction in charge repulsion observed in MD simulations.

### Trp65 is crucial in the molecular link between the trimeric units of CotE

Based on the AlphaFold2-predicted model and the cryo-EM map, the Trp-mediated binding interface demonstrated a strong potential for inter-trimer interactions, with experimental results supporting the central role of Trp65 in the binding interface. To disrupt these interactions, we introduced a W65E mutation in CotE, replacing Trp65 with glutamate, thereby creating charge repulsion at the center of the binding interface and effectively disrupting Trp-mediated interactions.

The resulting W65E variant CotE protein in its C-terminal His-tagged form was purified to homogeneity. The W65E-mutant protein exhibited a significantly smaller molecular weight (~61 kDa) in SEC than the wild-type CotE, irrespective of the presence of Ca^2+^ (Fig. 3e). Multiangle light scattering (MALS) analysis confirmed a molecular weight of approximately 70 kDa for the W65E CotE variant, which aligned with that of the CotE homotrimer (Fig. S12). The results of the thermal shift assay, shown in Fig. S13, revealed that the W65E-mutant of CotE was correctly folded, with a thermal transition exceeding 90°C, similar to that of wild-type CotE. Coupled with the high thermal stability of the W65E variant, our results indicated that CotE inherently forms stable trimeric units as the fundamental soluble unit. These results support the AlphaFold2-predicted model, highlighting the pivotal role of Trp65 in CotE oligomerization and molecular mesh assembly.

### Building a 3D model of the CotE meshwork in a diamond-like configuration

**Fig 4:**
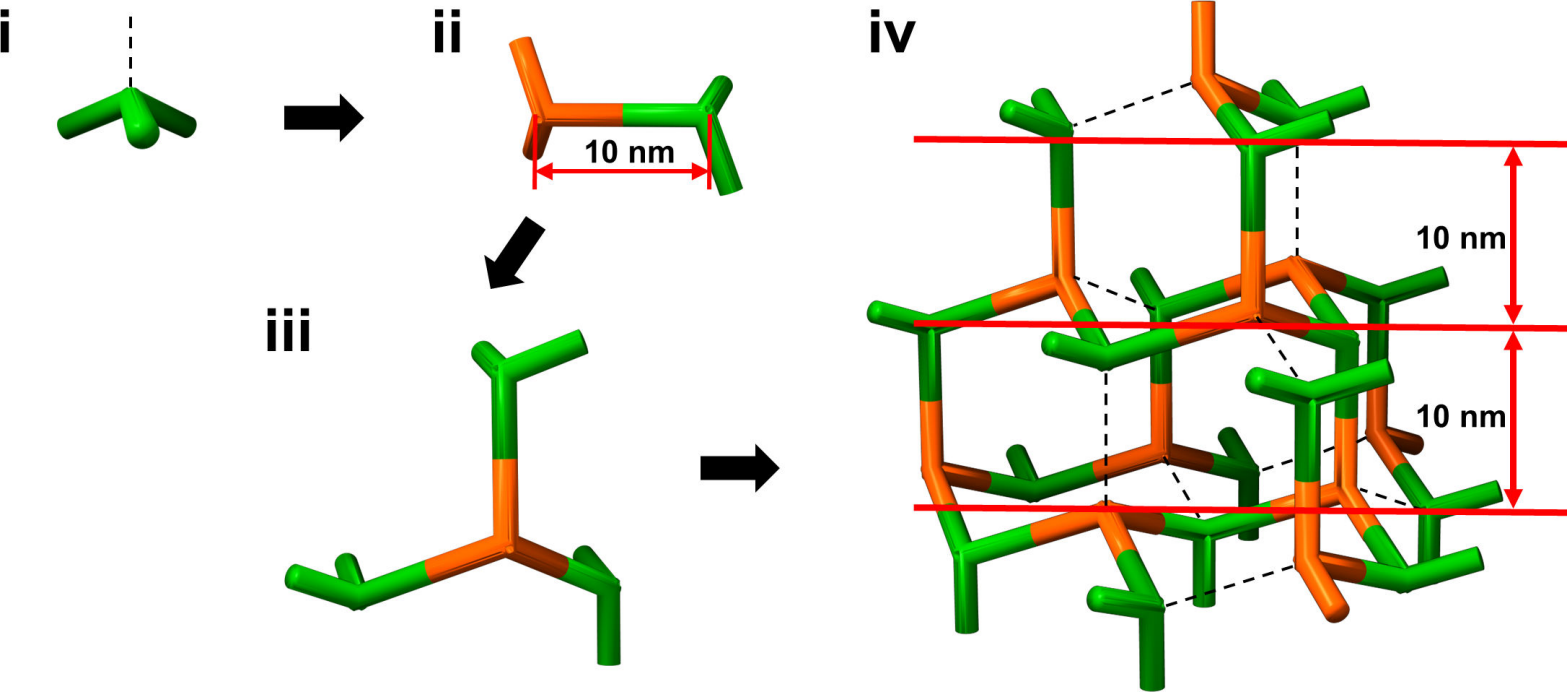
Schematic representation of the predicted structural model of the CotE protein The schematic illustrates the stepwise assembly forms of the CotE trimer model, progressively increasing in size. In step i, the trimer model of CotE is depicted, assuming a tetrapod structural conformation. The three thick green pillars represent the N-terminal body of each CotE monomer, while the black dashed lines indicate the hypothetical axes formed when the three additional CotE pillars are part of a tetrapod. The formation of a tetrahedral shape is demonstrated by linking the vertices to each pillar. Step ii presents the hexameric assembly model, in which two CotE trimers—shown in green and orange—are assembled. The two trimers are twisted 60° relative to each other rather than being connected in parallel. The spacing between each trimer center is expected to be 10 nm, based on the assembly model of the CotE protein. Step iii illustrates the model created by combining four CotE trimers. Step iv shows the tetrapod-like shape of the CotE trimer when forming the 3D assembly structure. The virtual axes depicted by black dashed lines in step i are interconnected to form a 3D grid, consisting of layers with an approximate gap of 10 nm between each layer.

Considering the cryo-EM model consisting of two trimers with a torsion angle of 60°, we constructed a 3D model of the CotE meshwork. This model incorporates inter-trimer interactions facilitated by Trp65, involving a 60° rotation around the axis between the trimer centers. As these trimeric units link, they form a meshwork, with each unit undergoing a 60° rotation about the connecting axis. This rotational motion transitioned the trimer from a flat arrangement to a three-dimensional configuration, as illustrated in Fig. 4. The 3D mesh structure formed had a height of approximately 10 nm per layer. Our observations revealed a hypothetical similarity between the 60° torsion angle connections of the trimers and the tetrahedral geometry of diamonds (Fig. S14). The center of the CotE trimer was aligned with the tetrahedral center of the carbon atoms in diamonds. However, unlike the carbon atoms in a diamond, the CotE trimer features only three arms, one fewer than the carbon structure of a diamond. Consequently, we describe the unique structure observed in the CotE meshwork as a defective diamond-like arrangement that potentially endows the CotE meshwork with subtle structural flexibility.

### Cryo-ET structure of the CotE 3D meshwork

**Fig 5:**
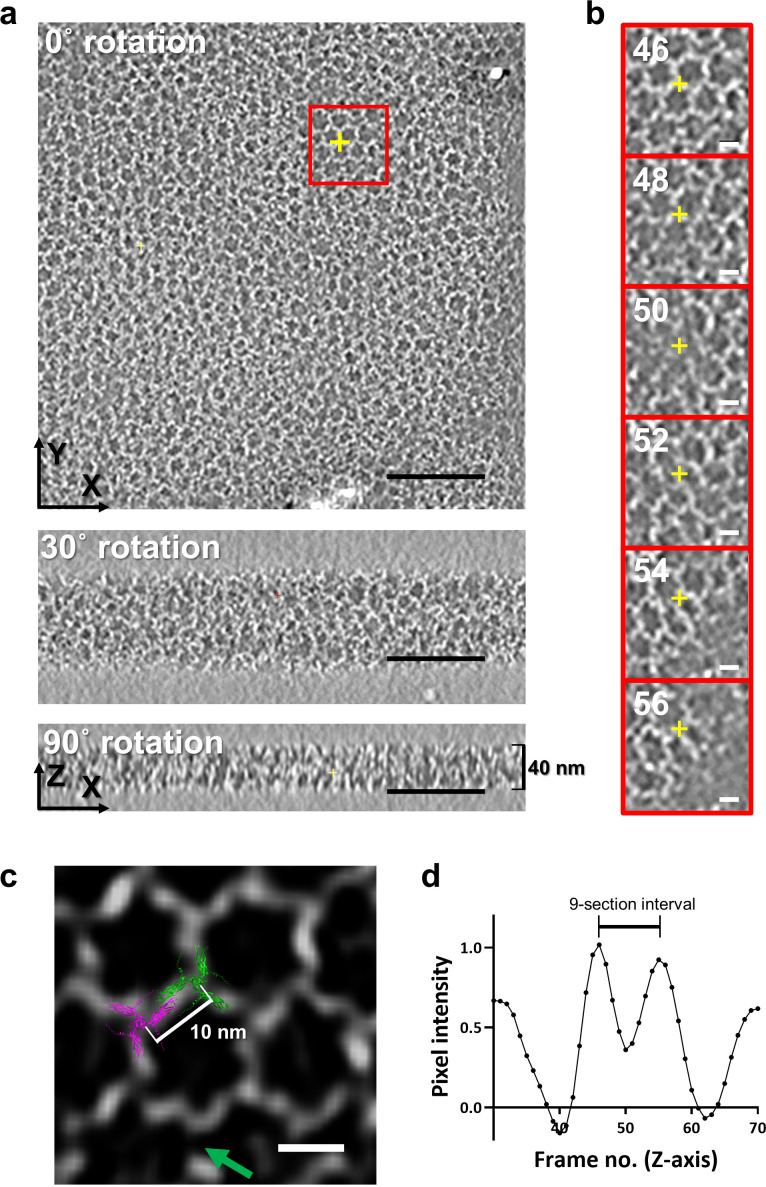
Cryo-ET structure of the CotE 3D meshwork. (**a**) Rotational views of the cryo-ET tomogram of *Bacillus cereus* CotE protein at angles of 0° (Frame No. 46), 30°, and 90°. The mesh-like structure of the CotE protein remains visible at tilted angles. The black scale bar represents 100 nm. (**b**) The area marked with a red box in Fig. 5a was enlarged and displayed at two-frame intervals. The central mesh structure disappears and reappears over approximately 10 tomogram sections. The white scale bar represents 10 nm. (**c**) The dual CotE trimeric model (magenta and green), predicted by AlphaFold2, was superimposed on the mesh structure of the CotE protein in the central area of the red box in panel b (top). The green arrow indicates the defect in the tetrahedral configuration of the 3D mesh. The white scale bar represents 10 nm, indicating that the distance between the centers of each trimer is 10 nm. (**d**) The variation in pixel intensities at the coordinates (*x* = 517, *y* = 435), marked by the yellow cross in panels a and b, was observed along the *Z*-axis sections representing the CotE mesh layer (30th–70th sections). An interval of approximately 10 sections (corresponding to 10 nm) was noted along the *Z*-axis. A similar pattern of 10 nm periodicity in different areas of the image is presented in Fig. S16.

To better define the 3D structure of the CotE meshwork, we performed cryo-ET with grids prepared using the same method as that used for cryo-EM, without ion-beam milling. We collected cryo-ET tomogram images from the data set with ±60° tilting angles (Fig. S15; Movie S1). The images show an expanded honeycomb-like pattern from selected representative Quantifoil holes (Fig. 5a). The processed tomogram consisted of 100 sections along the *Z*-axis, each with a thickness of 1 nm (Fig. S15a). The overall mesh structure of CotE was flat and embedded in the ice layer, observed from the 30th to the 70th sections, indicating a mesh thickness of approximately 40 nm (Fig. 5a), which matches the outer coat thickness of the endospores (9). Each tomogram section exhibits continuous changes in the size and position of the hexagonal lattice, with frequent defects (Fig. 5b and c). It was evident from the cryo-EM observation that the hexagonal lattices on the 2D *XY* plane were composed of a trimeric model, with dimers joining between the arms of the neighboring trimers (Fig. 5c). The distance between the centers of the trimers was 10 nm (Fig. 5c), which matched the cryo-EM models of the 3D meshwork.

By tracing specific regions across the *Z*-sections and analyzing the tomograms, we found that the hexagonal pattern reappeared at intervals of approximately 10 sections (Fig. 5b; Fig. S16). In addition, as shown in Fig. 5d, tracking the intensity of a specific pixel revealed that the intensity peaks appeared at intervals of approximately 10 nm. These results indicate that the trimeric centers were repeated at intervals of 10 nm along the *Z*-axis. Consequently, the periodicity of the trimers was consistent along both the 2D *XY* plane and the *Z*-axis. This uniform periodicity along the *X*-, *Y*-, and *Z*-axes resembles a diamond-like tetrahedral geometry, as proposed by the 3D model based on cryo-EM structures. Despite the resolution limitations of the cryo-ET structure, which caused the tilting projection images to be unclear, a similar defective hexagonal lattice pattern was observed at a tilting angle of 30° (Fig. 5a). In conclusion, the model of the defective diamond-like configuration was well supported by this cryo-ET observation, although higher-resolution structures might be required to demonstrate the meshwork structure at the atomic level.

### Role of Ca^2+^ and the C-terminal tail of CotE

**Fig 6:**
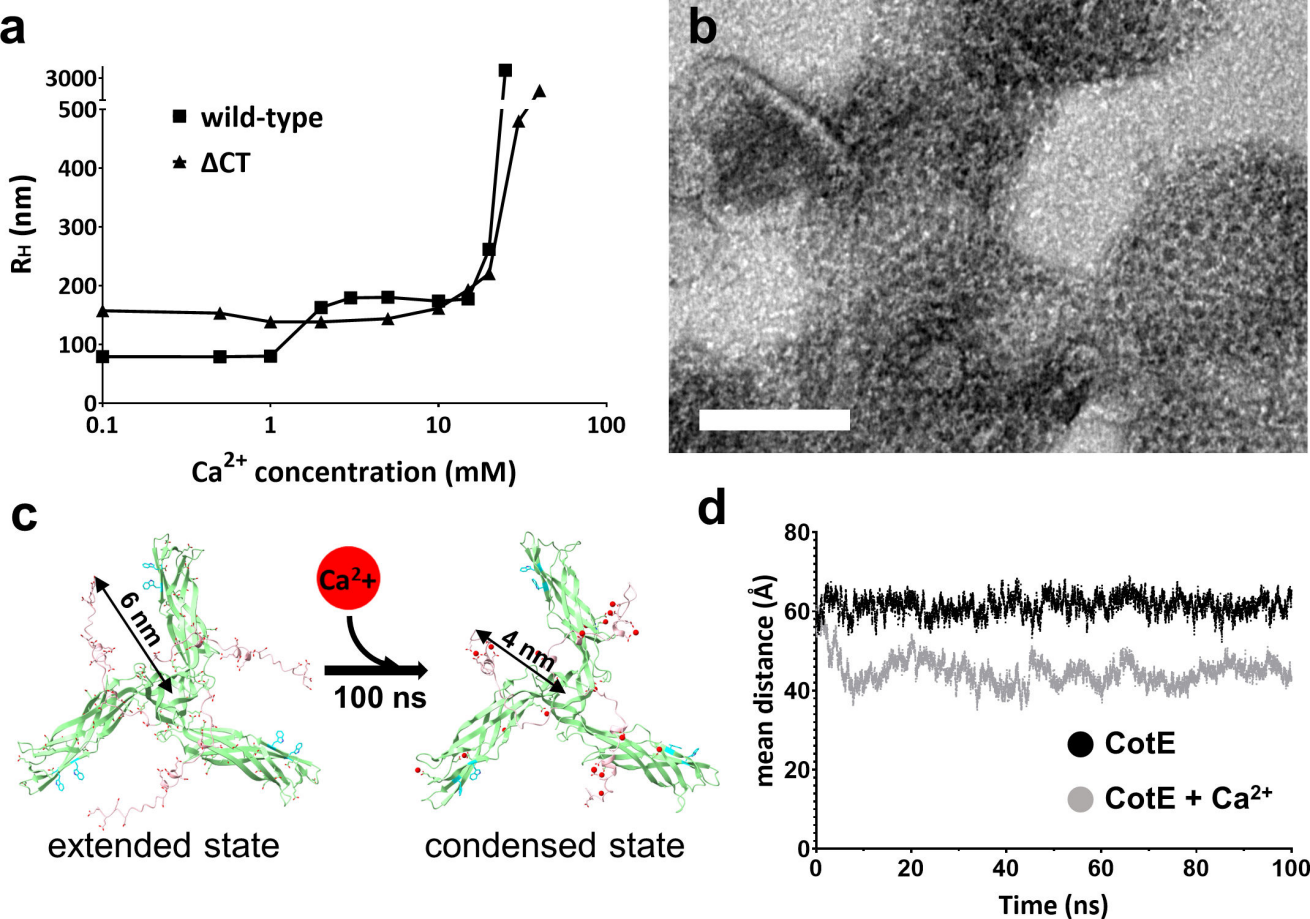
Role of the C-terminal unstructured tail. (**a**) Hydrodynamic radius (R_H_) changes of the ΔCT-CotE protein in response to Ca^2+^ using dynamic light scattering. Wild-type CotE protein was used as a control (Fig. 1b). The square mark represents R_H_ for the wild-type CotE protein, and the triangle mark represents that of the ΔCT-CotE protein. (**b**) A negative-stain electron microscopy image of the ΔCT-CotE protein in the absence of Ca^2+^. The scale bar indicates 100 nm. (**c**) The molecular dynamics (MD) simulation of the wild-type CotE protein in the presence of 5 mM Ca^2+^. The two structural models depict the wild-type CotE protein before and after undergoing a 100 ns MD simulation in the presence of 5 mM Ca^2+^. Only bound Ca^2+^ ions are displayed in the figure. The protein model utilized the predicted structure generated by AlphaFold2, with the N-terminal body domain depicted in green and the C-terminal tail in pink. Red circles denote the bound Ca^2+^ ions. The MD simulation result without Ca^2+^ is shown in Fig. S22b. (**d**) Distance plots from the MD simulation of the CotE protein both with and without Ca^2+^. The root-mean-squared distance between the trimeric center (Val145) and the C-terminal end (Glu180) is monitored using MD simulation for 100 ns, both in the absence and presence of 5 mM Ca^2+^.

To understand the function of the C-terminal unstructured tail of CotE, we constructed a mutant (ΔCT-CotE, residues 1–156) that lacks the C-terminal tail. DLS and negative-stain EM demonstrated that, in contrast to the full-length protein, which requires 5 mM of Ca^2+^ to form a meshwork, the ΔCT-CotE protein forms a honeycomb-like meshwork without Ca^2+^ (Fig. 6a and b). Consistently, the R_H_ of ΔCT-CotE remained unchanged across a Ca^2+^ concentration range of 0–20 mM, similar to the R_H_ of the full-length protein in the presence of 5 mM Ca^2+^. Negative-stain EM analysis of the ΔCT-CotE protein performed in the absence of Ca^2+^ showed a similar meshwork, demonstrating that the C-terminal tail region is responsible for the Ca^2+^ dependence in meshwork formation. These findings suggest that the C-terminal tail acts as a key element in Ca^2+^-dependent meshwork formation by counteracting the inhibitory effects of the C-terminal region.

To elucidate the inhibitory role of the C-terminal region and the counteracting role of Ca^2+^ in CotE meshwork assembly, we employed molecular dynamics (MD) simulations using GROMACS (24). During the 200 ns MD simulations, the terminal segment of the C-terminal tail region, containing four glutamic acid residues, remained in proximity to Trp65 with an extended conformation (Fig. 6c). In the presence of 5 mM Ca²^+^, several Ca²^+^ ions were attached to the negatively charged residues in the C-terminal and N-terminal domains of the trimeric core, suggesting a potential mechanism by which a conformational shift occurs from an extended to a condensed state of the C-terminal region (Fig. 6d; Fig. S22c).

The mechanism by which the C-terminal tail inhibits interactions between trimers in their extended states remains unclear. We propose that the highly negatively charged C-terminal tails electrostatically repel each other, preventing close contact between the trimers. Experimental observations and MD simulations indicate that upon Ca²^+^ binding, the condensed C-terminal tails likely undergo electrostatic neutralization, eliminating charge repulsion and facilitating inter-trimer binding. These simulation results propose a potential hypothesis for how Ca²^+^ may modulate the function of the C-terminal tails to regulate inter-trimer interactions.

### DPA or CaDPA-dependent disassembly of the Ca-CotE meshwork

**Fig 7:**
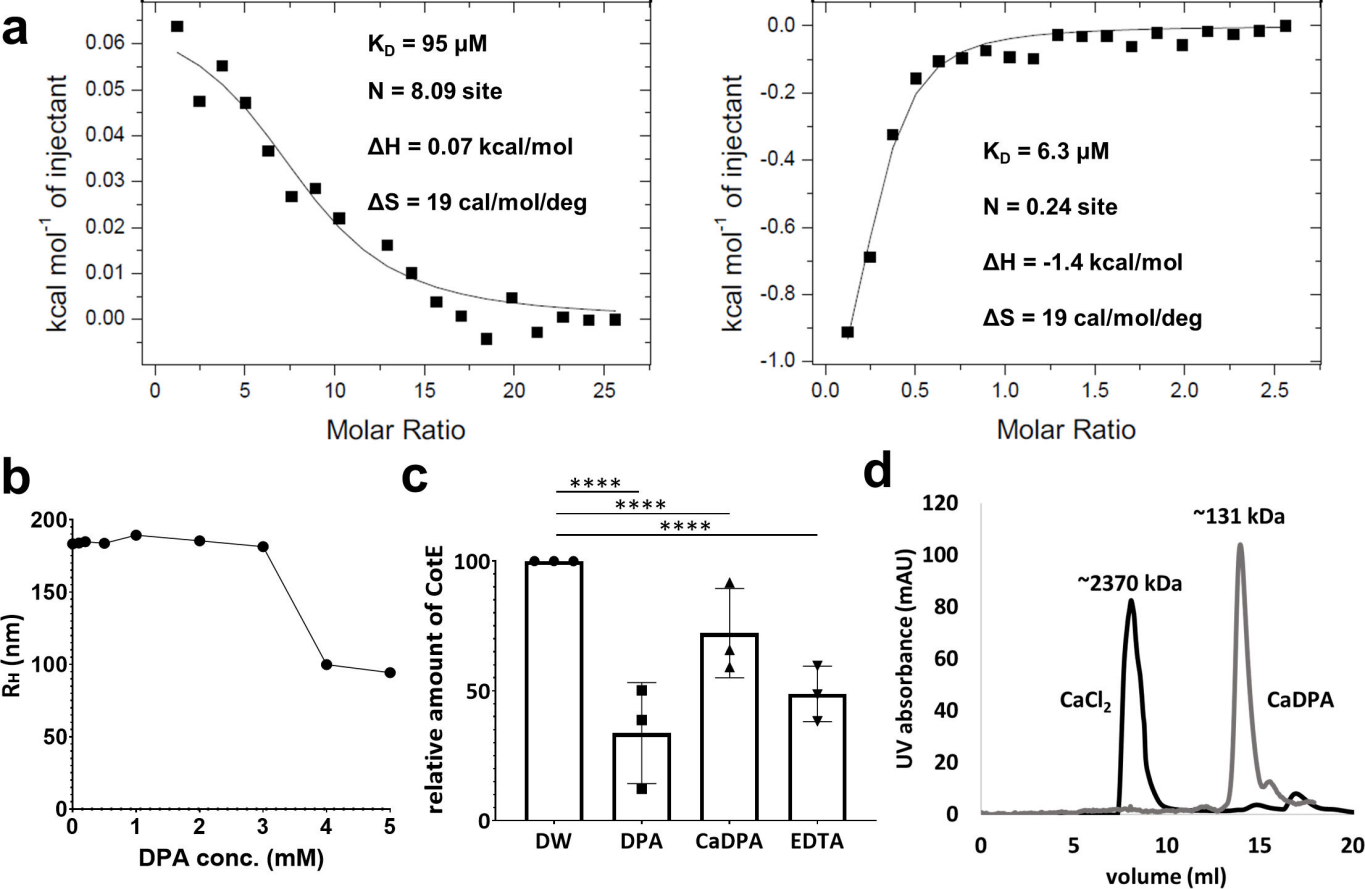
Effect of DPA on the structural transition of the CotE meshwork. (**a**) Fitting plots for the ITC. The W65E-mutant CotE proteins (100 µM) in PBS were placed in the sample cell, and either 10 mM CaCl_2_ (left) or 1 mM DPA (right) was placed in the syringe as the titrant. The thermodynamic parameters for the binding of the protein to Ca^2+^ or DPA are displayed in each diagram. Calorimetric titration profiles for the fitting curves can be found in Fig. S23. (**b**) Change in the hydrodynamic radius (R_H_) of the Ca-CotE meshwork in response to DPA concentration. Ca-CotE protein was dissolved in a 20 mM Tris (pH 8.0) buffer containing 5 mM CaCl_2_, with varying concentrations of DPA ranging from 0 to 5 mM. R_H_ values were measured using dynamic light scattering. (**c**) Amount of precipitated protein remaining without being resolubilized when each chelator (DPA, CaDPA, and EDTA) is used. A total of 50 mM Ca^2+^ was added to 1 mg CotE protein in the buffer to precipitate the protein, and then the buffer was exchanged for one containing 5 mM chelators to re-solubilize the precipitated CotE. Deionized water was utilized alongside the chelators as a control. The resolubilized protein was removed, and the amount of protein in the remaining pellet was analyzed three times by SDS-PAGE. Differences were considered statistically significant at *P*-values < 0.05. Asterisks (****) denote *P* < 0.0001. (**d**) SEC analysis of CotE at a concentration of 5 mM CaCl_2_ and 5 mM CaDPA. Two chromatograms are shown: the CotE protein (5 mg/mL) in SEC buffer with 5 mM CaCl₂ is depicted with a black line, while the CotE protein in the presence of 5 mM CaDPA is represented by a gray line. Note that the SEC profile for the 5 mM CaCl_2_ added CotE profile is reproduced from Fig. 1C. Peaks corresponding to different forms of CotE are annotated with their estimated molecular weights calculated using the protein size reference shown in Fig. S20.

To evaluate the binding affinity of CotE for Ca^2+^ and DPA, we measured the dissociation constants (*K*_*D*_) using isothermal titration calorimetry (ITC). The ITC results for wild-type CotE were inconclusive because of excessive noise (Fig. S17); therefore, we utilized the W65E CotE mutant, whose oligomeric state was not altered by Ca^2+^ or DPA (Fig. S18). The ITC results for the W65E-mutant (Fig. 7a) showed a *K*_*D*_ of 95 µM for Ca^2+^ and 6.3 µM for DPA. In particular, eight Ca^2+^-binding sites were identified in the CotE molecule, demonstrating the high Ca^2+^-binding capacity of CotE at the tested Ca^2+^ concentration. Given the measured *K*_*D*_ values of DPA, DPA should bind to CotE during germination, during which a high concentration of CaDPA is released from the spore core (25).

We further investigated how DPA affects the molecular events of Ca-CotE during germination. When Ca-CotE was treated with 0.1–5 mM DPA, its R_H_ (measured by DLS) decreased in a manner that reversed the changes seen with Ca^2+^ addition (Fig. 7b). To understand the effects of DPA, we compared them with those of EDTA and CaDPA. Figure 7c shows that DPA was more effective than EDTA, a stronger Ca^2+^ chelator, in solubilizing Ca-CotE, which precipitated into the soluble pool. Interestingly, Ca-CotE also became soluble in CaDPA, even though CaDPA did not sequester Ca^2+^, although its effect was weaker than that of DPA and EDTA. These findings indicate that as CaDPA cannot bind to Ca^2+^, the effect of DPA on CotE is not solely due to Ca^2+^ sequestration. Furthermore, SEC analysis revealed that the molecular size of Ca-CotE decreased significantly in a buffer containing 5 mM CaDPA, although it remained larger than that of the trimeric W65E-mutant protein (Fig. 7d and 3e). Since CaDPA did not affect the trimeric W65E protein in SEC, it was inferred that CaDPA interferes with the interactions between CotE trimers. Therefore, it appears that both DPA and CaDPA disrupted the molecular interactions in the Ca-CotE network, dismantling the trimeric structure.

### Encapsulation of proteins by the Ca-CotE hydrogel meshwork

**Fig 8:**
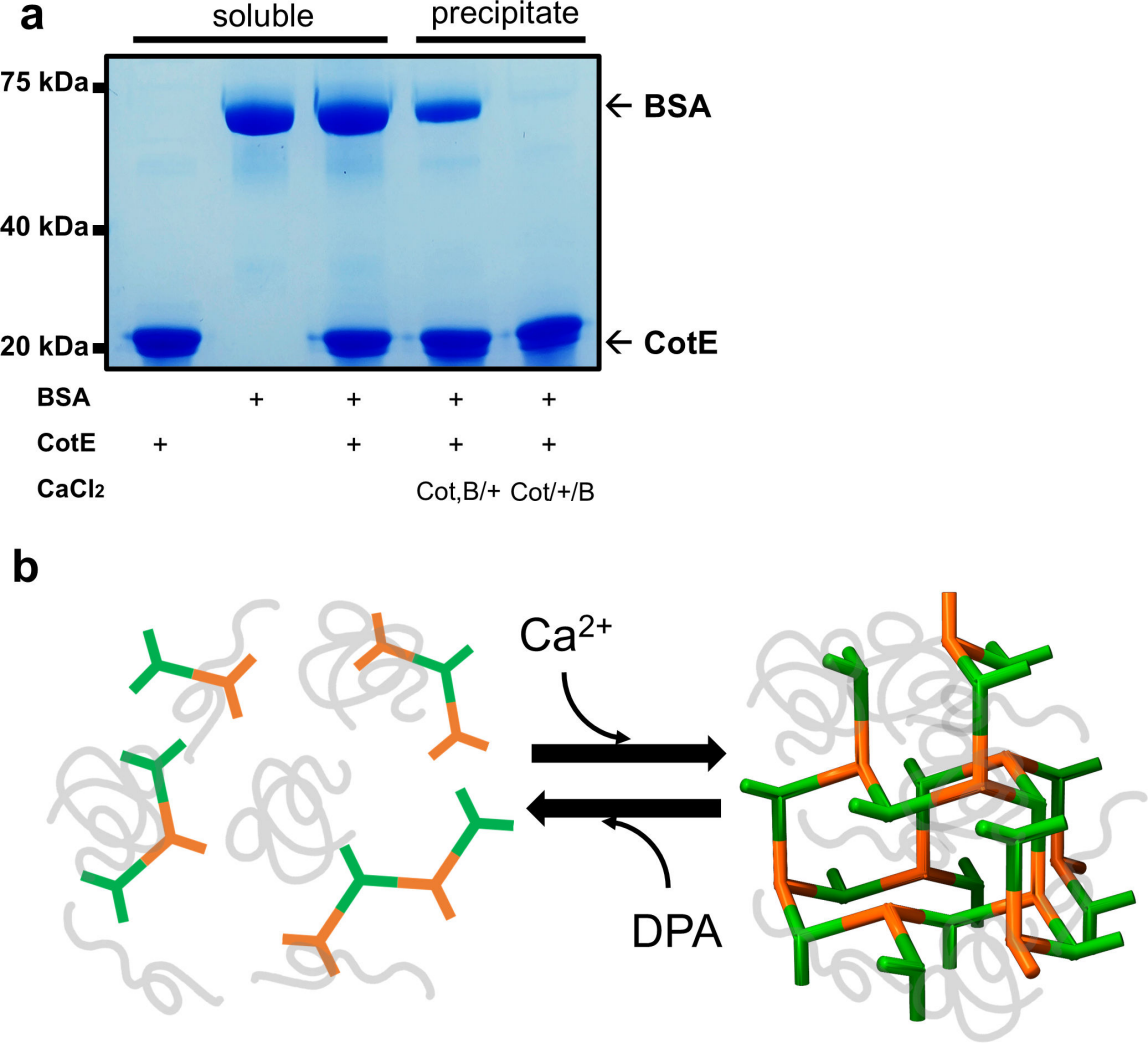
3D porous network structure of CotE, regulated by Ca^2+^ and DPA. (**a**) Evaluation of the relative retention of BSA protein after washing the pellet during Ca^2+^ precipitation. Sodium dodecyl sulfate-polyacrylamide gel electrophoresis (SDS-PAGE) was conducted to assess the protein content under various conditions. Columns 1, 2, and 3 represent SDS-PAGE results for CotE alone, BSA alone, and a CotE-BSA mixture, respectively. These columns illustrate the protein positions on SDS-PAGE and their concentrations upon mixing. Columns 4 and 5 present SDS-PAGE results of CotE precipitates after Ca^2+^ precipitation (50 mM). In column 4, precipitation occurred upon mixing CotE and BSA, while in column 5, BSA was mixed after CotE precipitation by Ca^2+^. Upon washing the precipitate and allowing it to settle, column 4 retained up to 50% of the BSA, while column 5 showed a complete absence of BSA. (**b**) Proposed pathways between the soluble CotE and the oligomeric state. In its initial phase, CotE protein fragments exist in a soluble state. Upon the introduction of Ca^2+^, these molecular fragments undergo self-assembly, forming a diamond-shaped 3D lattice that encapsulates the proteins within its structure. The addition of DPA triggers a reverse transformation, facilitating the disassembly of the CotE aggregate into its soluble fragments, thereby releasing the encapsulated proteins.

Hydrogels tend to encapsulate proteins in solution. Thus, we explored the hydrogel-like characteristics of the Ca-CotE meshwork, utilizing bovine serum albumin (BSA) as a representative model protein. Given that BSA is a highly soluble protein with a molecular mass of 67 kDa, it may serve as a proxy for the outer coat protein, CotA, which has a molecular mass of 57 kDa. Upon the addition of Ca^2+^ to a mixture containing CotE and BSA, the resultant Ca-CotE aggregates were observed to encapsulate a significant quantity of BSA in sodium dodecyl sulfate-polyacrylamide gel electrophoresis (SDS-PAGE) (Fig. 8a, lane 4). Conversely, the incubation of BSA with preformed Ca-CotE aggregates resulted in no BSA incorporation (Fig. 8a, lane 5). These findings demonstrate that CotE can encapsulate soluble BSA by forming a hydrogel in the presence of Ca^2+^. This further implies that once the Ca-CotE meshwork is formed, it becomes impermeable to proteins of 67 kDa or more. However, the permeability threshold of the Ca-CotE mesh remains undetermined.

## DISCUSSION

The endospore coat provides a highly resistant barrier against physical and chemical stress. These spores are designed to germinate and resume vegetative growth under favorable conditions. We observed that the 3D meshwork of CotE was induced by Ca^2+^ in relation to its C-terminal tail. Cryo-EM, cryo-ET, and AlphaFold2 predictions demonstrated that CotE formed a hydrogel architecture through a unique, defective diamond-like connection. We highlighted the crucial role of Trp65 in CotE oligomerization, which is essential for organizing the 3D meshwork. This 3D meshwork could trap and encapsulate other proteins in its porous structure. Furthermore, we observed that DPA disrupted the CotE meshwork, providing molecular insights into how this robust layer was quickly dismantled during germination.

We discovered that Mn²^+^ and Ni²^+^, along with Ca²^+^, induced the formation of a 3D meshwork of CotE at a concentration of 5 mM but not at 0.1 mM (Fig. S1). However, Mn²^+^ and Ni²^+^ are unlikely to reach millimolar concentrations under typical environmental conditions (26). Therefore, we ruled out the physiological relevance of Mn²^+^ and Ni²^+^ in CotE meshwork formation, while Ca²^+^ remains a likely factor due to its physiological abundance. Can Ca^2+^ reach the millimolar levels necessary to form a molecular network with CotE? Direct deposition of Ca^2+^ onto the outer coat during encasement has not yet been experimentally confirmed. However, the P-type Ca^2+^-importing transporter *yloB* in *Bacillus* cells is expressed by the σ^F^ factor in the mother cells before forming the coat layers (27). These findings suggest that Ca^2+^ could be supplied to the outer coat from the surrounding medium or environment, such as soil, which contains 1–10 mM Ca^2+^ (28). Supporting this idea, *Bacillus* endospore formation was enhanced in a Ca^2+^-rich medium (29). Importantly, a significant amount of DPA is synthesized in the mother cell and transported as CaDPA into the forespore core before the formation of coat layers (18). This suggests that free Ca^2+^ levels in mother cells may increase once DPA synthesis ceases. Therefore, it is reasonable to infer that Ca^2+^ concentrations in the spore coat layers could reach millimolar levels as sporulation progresses, though further experimental evidence is required to confirm this.

The honeycomb-like arrangement of Ca-CotE was observed using negative-stain EM, as illustrated in Fig. 1d. The diamond-like arrangement in space was transformed into a planar honeycomb-like arrangement on the surface by adjusting the torsion angles to parallelize the two symmetrical axes of the trimeric and dimeric interactions, as shown in Fig. S19. The resulting planar honeycomb-like arrangement was consistent with the negative-stain EM images. The drying process of carbon thin films is likely to cause potential distortions in the meshwork during negative-stain EM procedures.

We propose a mechanistic pathway for the encasement of the outer coat, designed to form a compact and impermeable layer that rapidly disintegrates at the onset of germination (Fig. 8b). Initially, the soluble trimeric unit of CotE is localized outside the inner coat through specific interactions with SpoVID (30). As sporulation progresses and Ca^2+^ or divalent ion levels increase, CotE forms a 3D meshwork through dimeric interactions between the CotE trimers and the outer coat proteins (Fig. 8b, right). A recent cryo-ET study of nascent coat layers in *B. subtilis* revealed CotE-dependent layers composed of bead-like objects observed midway through engulfment, partially covering the forespore pole (11). The size of these bead-like objects (approximately 8 nm) aligns with the radius of the CotE trimer, suggesting that this CotE-dependent layer represents one layer of the assembly of trimeric CotE units. We speculate that this CotE-dependent layer, characterized by bead-like features, could form an interface between the inner coat or the exosporium (or crust) layer of the endospore.

During germination, upon encountering DPA, the outer coat layer disintegrates due to the loss of molecular interaction with CotE, leading to the rapid expansion of the compact structure. This mechanism underlies dynamic structural adjustments in the spore coat in response to environmental stimuli, highlighting the intricate balance between spore dormancy and germination readiness.

In the present study, we investigated the molecular structure of CotE using a combination of experimental and computational approaches. Our findings regarding the formation and breakdown of the CotE meshwork shed light on the molecular mechanisms underlying the encasement and germination of endospores. These findings will help develop new ways to combat bacteria that are resistant to physical and chemical stresses during sterilization processes.

## MATERIALS AND METHODS

### Plasmid construction and expression of the CotE proteins

Wild-type CotE from *Bacillus cereus* was synthesized using gene synthesis services (Bioneer, Daejeon, Republic of Korea) with codon optimization for *Escherichia coli*. The synthesized CotE gene was inserted into the NcoI and XhoI sites of the pET28a vector (Merck, Madison, WI, USA), resulting in pET28a-CotE, which lacks a 6× His-tag at either terminus. To generate the W65E variant protein, a point mutation was introduced using the polymerase chain reaction (PCR)-based QuikChange method (31) with the primers listed in Table S1 and the recombinant pET28a vector, resulting in pET28a-CotE (W65E), which includes a 6× His-tag at the C-terminus. The C-terminal tail-deleted (ΔCT) CotE variant gene (residues 1–156) was obtained via PCR using primers specific to pET28a-CotE and then inserted into the NcoI and XhoI sites of the pET28a vector, yielding pET28a-ΔCT-CotE, which also lacks a 6× His-tag at either terminus, similar to the wild type. The resulting expression vectors were transfected into *E. coli* BL21 (DE3) cells (Merck, Madison, WI, USA). The transformed cells were cultured in 1.5 L of LB medium supplemented with 50 μg/mL kanamycin at 37°C until reaching an OD_600_ of 0.6. Subsequently, protein expression was induced by adding 0.5 mM isopropyl-β-D-thiogalactoside to the culture medium. After an additional 6 h of incubation at 30°C, the cells were harvested by centrifugation at 5,500 × *g*.

### Purification of the wild-type and the C-terminal-deleted CotE proteins

The harvested *E. coli* cells expressing the wild-type and C-terminal-truncated CotE proteins were resuspended in 50 mL lysis buffer containing 20 mM Tris-HCl (pH 8.0). The resuspended cells were disrupted by sonication, and the resulting cell debris was removed by centrifugation at 20,000 × *g*. The remaining supernatant was passed through a Q anion-exchange chromatography column (5 mL HiTrap Q HP; Cytiva), and the CotE-containing unbound solution was precipitated by adding 50 mM CaCl_2_. Following centrifugation at 4,000 × *g*, the precipitate was collected and solubilized by adding 20 mM Tris-HCl buffer (pH 8.0) containing 200 mM freshly prepared DPA. The solubilized CotE was further purified using a HiPrep 16/60 Sephacryl S-300 HR column (Cytiva) with a 20 mM Tris-HCl (pH 8.0) buffer. The eluted fractions of CotE proteins were finally purified using a HiLoad 16/600 Superdex 200 pg (Cytiva) column with the same buffer mentioned above.

### Purification of the CotE W65E-mutant protein

Harvested cells expressing the W65E-mutant CotE were resuspended in 50 mL lysis buffer composed of 20 mM Tris-HCl (pH 8.0) and 150 mM NaCl. The resuspended cells were subjected to sonication for disruption, and the resulting cell debris was removed by centrifugation at 20,000 × *g*. The His-tagged protein was purified using 1 mL of Ni-NTA agarose resin (Qiagen, Hilden, Germany). The CotE-bound resin was washed with lysis buffer supplemented with 20 mM imidazole (pH 8.0) and eluted with 15 mL 20 mM Tris-HCl (pH 8.0) buffer containing 150 mM NaCl and 250 mM imidazole (pH 8.0). Subsequently, Q anion exchange chromatography (5 mL HiTrap Q HP; Cytiva TM) was performed on the elution fractions using a NaCl gradient (0–1 M). The collected CotE-containing fractions were concentrated to 5 mL using a Vivaspin centrifugal concentrator (30 kDa molecular weight cutoff; Millipore, Hayward, CA, USA) and then applied to the SEC column HiLoad 16/600 Superdex 200 pg (Cytiva) in lysis buffer for final purification.

### DLS of the CotE proteins

Following dilution of the purified CotE proteins to approximately 1 mg/mL, CaCl_2_ and DPA were promptly added and thoroughly mixed by pipetting. The *Z*-averages were determined using a DLS instrument (Zetasizer Nano ZS90; Malvern Panalytical). The alteration in the *Z*-average was recorded by gradually introducing CaCl_2_ into the same cuvette, starting from concentrations of 0.5 mM and progressing up to 20 mM. Conversely, for the DPA experiments, the change in the *Z*-average was monitored by incrementally introducing DPA into the protein solution up to a concentration of 5 mM while maintaining a constant Ca^2+^ concentration of 5 mM.

### CotE-negative-TEM

Ten microliter of the CotE protein sample (0.02 mg/mL) was applied to 400-mesh carbon-coated copper grids (Electron Microscopy Sciences, EMS), which were freshly glow-discharged. The protein adsorbed on the grids was subsequently subjected to negative staining using a 1% uranyl acetate solution and then allowed to air dry at 25°C. Negative EM imaging was conducted using a 120 kV Tecnai G2 Spirit TWIN microscope (FEI, CMCI at SNU) equipped with a Rio 4 CMOS camera (Gatan).

### Cryo-EM of wild-type CotE

For the wild-type CotE, a 3 µL aliquot of the purified CotE solution (2 mg/mL), containing 5 µM CaCl_2_ (mixed just before application), was applied to glow-discharged COS grids (32) composed of holey-carbon Quantifoil R1.2/1.3, 300 mesh grids. These grids were blotted for 5 s with blot force 0 at 4°C and subsequently plunge-frozen in liquid ethane using a Vitrobot Mark IV system (Thermo Fisher Scientific Inc., USA). Cryo-EM micrographs were acquired using a 200 kV Glacios cryo-TEM microscope (Thermo Fisher Scientific) equipped with an X-FEG, a Ceta 16M camera, and a Falcon 4 camera. A total of 1,412 movies were collected at a nominal magnification of ×92,000 (resulting in a pixel size of 1.1 Å) and a total dose rate of 50 e Å^−2^ using automatic data acquisition software (EPU, Thermo Fisher Scientific Inc., USA). An exposure time of 14.77 s was employed, and the resulting videos were saved in the MRC format. A defocus range of −2.0 to −1.0 μm was utilized.

### Data processing and model building of wild-type CotE cryo-EM images

The CotE cryo-EM data set was processed using CryoSPARC (33). A total of 1,412 movies were imported into the CryoSPARC server, and the contrast transfer function of the prepared micrographs was estimated. From the initial data set, a manual classification step was performed to select images displaying the CotE network structure, which reduced the data set to 305 movies. Subsequently, approximately 300 Y-shaped CotE structures were manually selected, and 2D classification was performed to extract these images. Auto-picking was then conducted using a template picker, resulting in the identification of 304,159 particles. Further refinement involved selecting 2D classified images where the Y-shaped structure of CotE was recognizable, enabling the construction of a 3D electron density map using cryo-EM.

To construct a hexameric model of the CotE protein that accurately fits the cryo-EM map, two AlphaFold2-predicted trimer models were fitted to the C2-symmetry cryo-EM map using the *fitmap* function of ChimeraX.

### Computational prediction of CotE models

*B. cereus* outer spore coat protein CotE sequences were obtained from the National Center for Biotechnology Information (NCBI) server using the reference sequence WP_098905945.1. A series of predicted 3D models were generated using AlphaFold2. Specifically, AlphaFold-multimer version 2.3.0 was employed to predict models of the CotE protein in both homotrimer and homohexamer configurations. The majority of the settings were maintained at their default values; however, it should be noted that we utilized the multimer mode and reduced_dbs option to facilitate the generation of these models.

### MALS analysis of the CotE variant

High-performance liquid chromatography (HPLC) was performed using a Shimadzu system (Kyoto, Japan) equipped with a Superdex 75 Increase 10/300 Gl column (Cytiva). Chromatographic analysis was conducted at the KBSI Ochang Center in Ochang, Republic of Korea. The column was connected to a MALS instrument, which comprised a Wyatt DAWN Heleos II (with 18 angles of detection) and a Wyatt Optilab T-rex (refractive index detector) manufactured by Wyatt Technology (Goleta, CA, USA). For the HPLC analysis, the protein sample was prepared at a concentration of 2 mg/mL and loaded onto the column in a buffer containing 20 mM Tris (pH 8.0). Data analysis was performed using the ASTRA 6 software (Wyatt Technology, Goleta, CA, USA).

### MD simulation

The Gromacs software (34, 35) was used for MD simulations to evaluate CotE binding to Ca^2+^. The topology files for the protein and Gromacs files were prepared using the CHARMM36m force field in CHARMM-GUI (36, 37). The structure was solvated in transferable intermolecular potential with three points (TIP3P) water and neutralized by adding 10 mM Ca^2+^. Protein molecules were merged for each system, solvated with TIP3P water molecules, energy-minimized, and equilibrated. The system was subjected to energy minimization using the steepest descent algorithm. The deminimized state was equilibrated with a 100 ps NVT simulation to attain a temperature of 300 K and a 100 ps NPT simulation for a target pressure of 1 bar. The resulting complex structure was regarded as refined at 0 ns. The time step was set to 0.002 ps according to a published protocol. The MD simulation was performed for 100 ns, starting at the end of equilibration. The root mean square deviation of the simulated structure was calculated from the trajectory data using the Gromacs tool.

### ITC

ITC experiments were conducted to determine the binding affinity between the W65E-mutant CotE and Ca^2+^ or DPA. Experiments were performed using an Auto iTC200 Microcalorimeter (GE Healthcare) at the Korea Basic Science Institute (Ochang, Korea). W65E-mutant CotE proteins were prepared at a concentration of 100 μM in the sample cell (370 μL) and dialyzed against PBS (pH 7.2) overnight prior to the experiments. The ligands, Ca^2+^ or DPA, were dissolved in the same buffer at concentrations of 10 and 1 mM, respectively, and loaded into the injectable syringe (110 μL). The ITC experiments were conducted at 25°C while stirring the syringe at 750 rpm. The data were analyzed using the MicroCal Origin software.

### Size comparison of CotE variants using SEC

SEC was employed to assess the molecular size and potential oligomerization states of three proteins: wild-type CotE, W65E-mutant CotE, and ΔCT. For each protein, conformational changes were observed in the presence of additives such as Ca^2+^ and DPA. The analysis was conducted on a Superdex 200 Increase 10/300 GL SEC column (Cytiva). The buffer solution consisted of 20 mM Tris-HCl (pH 8.0) with the aforementioned additives. The apparent molecular weights of the proteins were determined based on their elution volumes using a calibration curve (Fig. S20).

### Re-solubilization efficacy comparison of three chelators

One milligram of CotE protein per milliliter in 20 mM Tris-HCl buffer (pH 8.0) was precipitated by adding 50 mM Ca^2+^, followed by centrifugation at 20,000 × *g* to remove the supernatant. Subsequently, 5 mM solutions of three different chelators (DPA, CaDPA, and EDTA) were prepared in a buffer containing 20 mM Tris (pH 8.0), and 100 µL of each solution was added to the precipitated CotE. The precipitated protein was then resolubilized through pipetting and centrifuged again at 20,000 × *g*. The supernatant fraction resolubilized by each chelator was collected, and the precipitated proteins were diluted to equal volumes for comparison via SDS-PAGE. This process was repeated three times, and the results obtained from SDS-PAGE were analyzed using the ImageJ tool (38).

### Confirmation of BSA encapsulation ability of CotE

Initially, a 100 µL buffer solution (20 mM Tris, pH 8.0) containing 1 mg each of CotE and BSA proteins (Bovostar, Bovogen, Australia) was treated with 50 mM Ca^2+^ and centrifuged at 20,000 × *g* to induce co-precipitation. The resulting co-precipitated complex was washed with a buffer (20 mM Tris, pH 8.0) supplemented with 50 mM Ca^2+^. In parallel, 50 mM Ca^2+^ was added to 1 mg of CotE protein dissolved in 50 μL buffer solution (20 mM Tris, pH 8.0), resulting in precipitation upon centrifugation at 20,000 × *g*. Subsequently, 1 mg of BSA dissolved in 50 μL buffer solution (20 mM Tris, pH 8.0) was combined with the precipitate and incubated. After incubation, the precipitate was washed with the aforementioned buffer. A comparative analysis of the protein content in the final pellets was performed using SDS-PAGE.

### Cryo-ET

A 3 µL aliquot of purified CotE solution (2 mg/mL), containing 5 mM CaCl_2_ was applied to a glow-discharged holey grid (Quantifoil R1.2/1.3 Cu300). The grid was plunge-frozen using a Vitrobot Mark IV (Thermo Fisher Scientific), with the chamber temperature and relative humidity set to 4°C and 90%, respectively. A blot force 5 was applied with blot and wait time of 5 and 0 s, respectively. Cryo-ET data were acquired using a Glacios transmission electron microscope operating at an acceleration voltage of 200 kV and equipped with a Falcon 4i direct electron detector (Thermo Fisher Scientific). The Tomography software (v5.16, Thermo Fisher Scientific) was used to collect images with tilt angles ranging from ± 60° with a 3° tilt increment. A nominal magnification of ×57,000, which corresponds to a pixel size of 1.83 Å at the specimen level, and a dose rate of 3.21 e^–^/Å^2^/s were applied. A defocus range between –2.0 and –3.0 μm was used for imaging. Tilt image stack data were processed using the AreFast software (v1.3.4) for automated tomographic reconstruction (39). The final tomogram was produced by binning with a factor of 4. The tomograms were subjected to denoising and missing wedge information recovery using the IsoNet software (v0.2) (40).

## Data Availability

Cryo-EM density maps of the trimeric and hexameric forms of *Bacillus cereus* CotE, along with the cryo-ET tomogram referenced in this study, have been deposited in the Electron Microscopy Data Bank (EMDB) under the following accession codes: EMD-63328 (trimer), EMD-63329 (hexamer), and EMD-63330 (tomogram). The data are accessible at https://www.ebi.ac.uk/emdb/.

## SUPPLEMENTAL MATERIAL

File S1 mbio.02472-24-s0001.pdf 464 BLASTp-aligned CotE sequences and their consensus sequence.

Supplemental material mbio.02472-24-s0002.pdf Figures S1 to S23 and Tables S1 and S2.

Movie S1 mbio.02472-24-s0003.mp4 Visualization of a representative BcCotE tomogram.

ASM does not own the copyrights to Supplemental Material that may be linked to, or accessed through, an article. The authors have granted ASM a non-exclusive, world-wide license to publish the Supplemental Material files. Please contact the corresponding author directly for reuse.
