# Supplementary figures and images for: 3D meshwork architecture of the outer coat protein CotE: implications for bacterial endospore sporulation and germination
Source: mBio. 2025 Mar 6;16(4):e02472-24. doi: 10.1128/mbio.02472-24 (PMC11980541; doi:10.1128/mbio.02472-24)

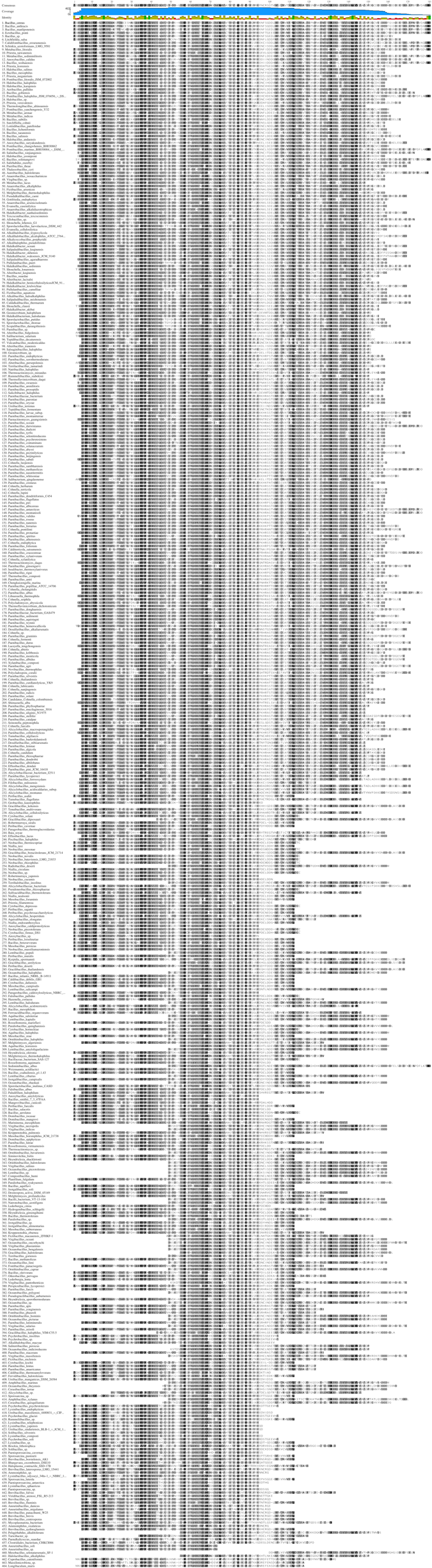

Supplement: File S1 — 464 BLASTp-aligned CotE sequences and their consensus sequence. [file mbio.02472-24-s0001.pdf]
