# Supplementary material for: 3D meshwork architecture of the outer coat protein CotE: implications for bacterial endospore sporulation and germination
Source: mBio. 2025 Mar 6;16(4):e02472-24. doi: 10.1128/mbio.02472-24 (PMC11980541; doi:10.1128/mbio.02472-24)
Supplement: Supplemental material — Figures S1 to S23 and Tables S1 and S2. [file mbio.02472-24-s0002.pdf]

SUPPLEMENTAL INFORMATION

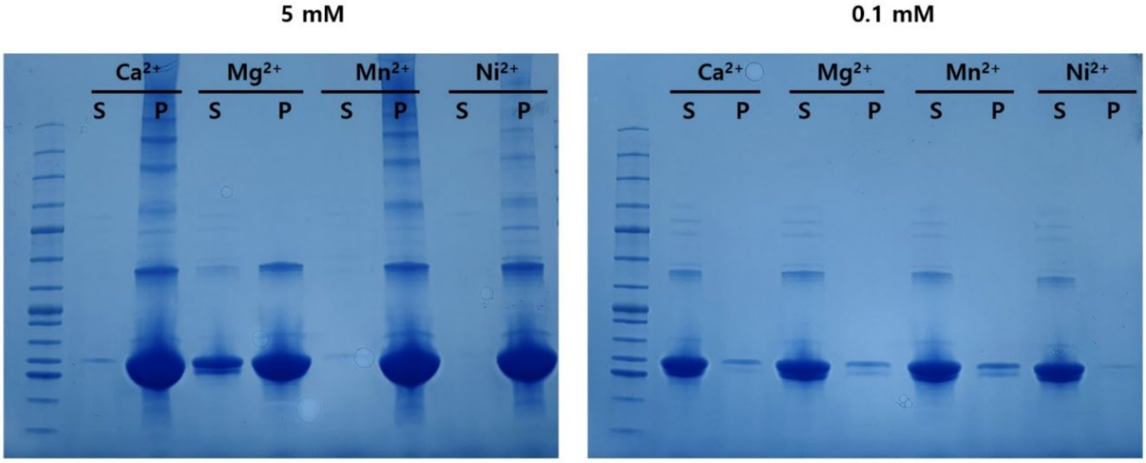

Supplementary Figure 1. Effects of diverse divalent ions on the precipitation of CotE protein

Wild-type CotE protein (1 mg/mL) was incubated in 20 mM Tris-HCl buffer (pH 8.0) at 25°C for 10 min, with either 5 mM (left gel) or 0.1 mM (right gel) of the indicated divalent ions: Ca^{2+} (as $CaCl_2$), Mg^{2+} (as $MgCl_2$), Mn^{2+} (as $MnCl_2$), and Ni^{2+} (as $NiCl_2$). After incubation, the samples were centrifuged at 15,000 rpm for 10 min at 4 °C. The resulting supernatant (S) and pellet (P) were analyzed by sodium dodecyl sulfate-polyacrylamide gel electrophoresis (SDS-PAGE).

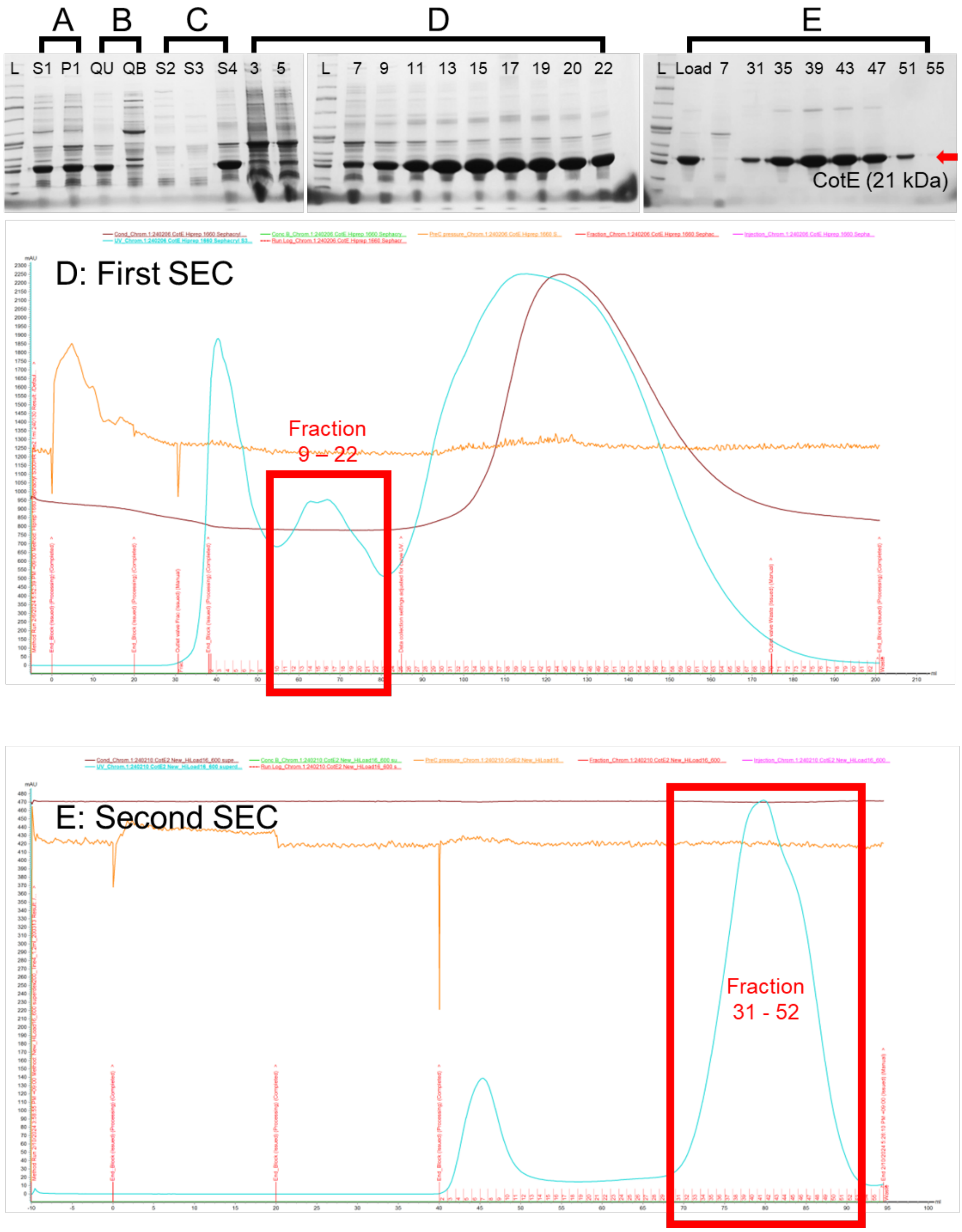

Supplementary Figure 2. Purification procedure of the BcCotE protein

Top: Representative sodium dodecyl sulfate-polyacrylamide gel electrophoresis (SDS-PAGE) gel images depicting the purification procedure for the *Bacillus cereus* CotE. (L indicates the protein size marker PM5100, with molecular weights of 245, 180, 140, 100, 75, 60, 50, 40, 35,

30, 25, 20, 15, and 10 kDa, from top to bottom). (A) Analysis of centrifuged *Escherichia coli* cell lysates overexpressing BcCotE, with S1 representing the supernatant fraction and P1 representing the pellet fraction. (B) Bound (QB) and unbound (QU) fractions were determined by Q-anion-exchange chromatography. (C) Ca^{2+} and DPA treatment of unbound fractions by Q-anion chromatography. S2 represents the supernatant fraction in a 50 mM $CaCl_2$-containing buffer. S3 represents the supernatant fraction of the precipitate obtained in the S2 step using the same buffer. S4 shows the DPA-solubilized fraction from the precipitate obtained in the S3 step. (D) Size-exclusion chromatography (SEC) results for the S4 fraction using a HiPrep 16/60 Sephacryl S-300 HR (Cytiva), with the elution profile shown in the middle panel. Fractions 9–22, highlighted with a red box in the elution profile, were collected. (E) Second SEC results using a HiLoad® 16/600 Superdex® 200 pg (Cytiva). “Load” refers to the sample loaded onto the SEC column, and fractions 31–52, highlighted by the red box in the second SEC elution profile in the bottom panel, were collected.

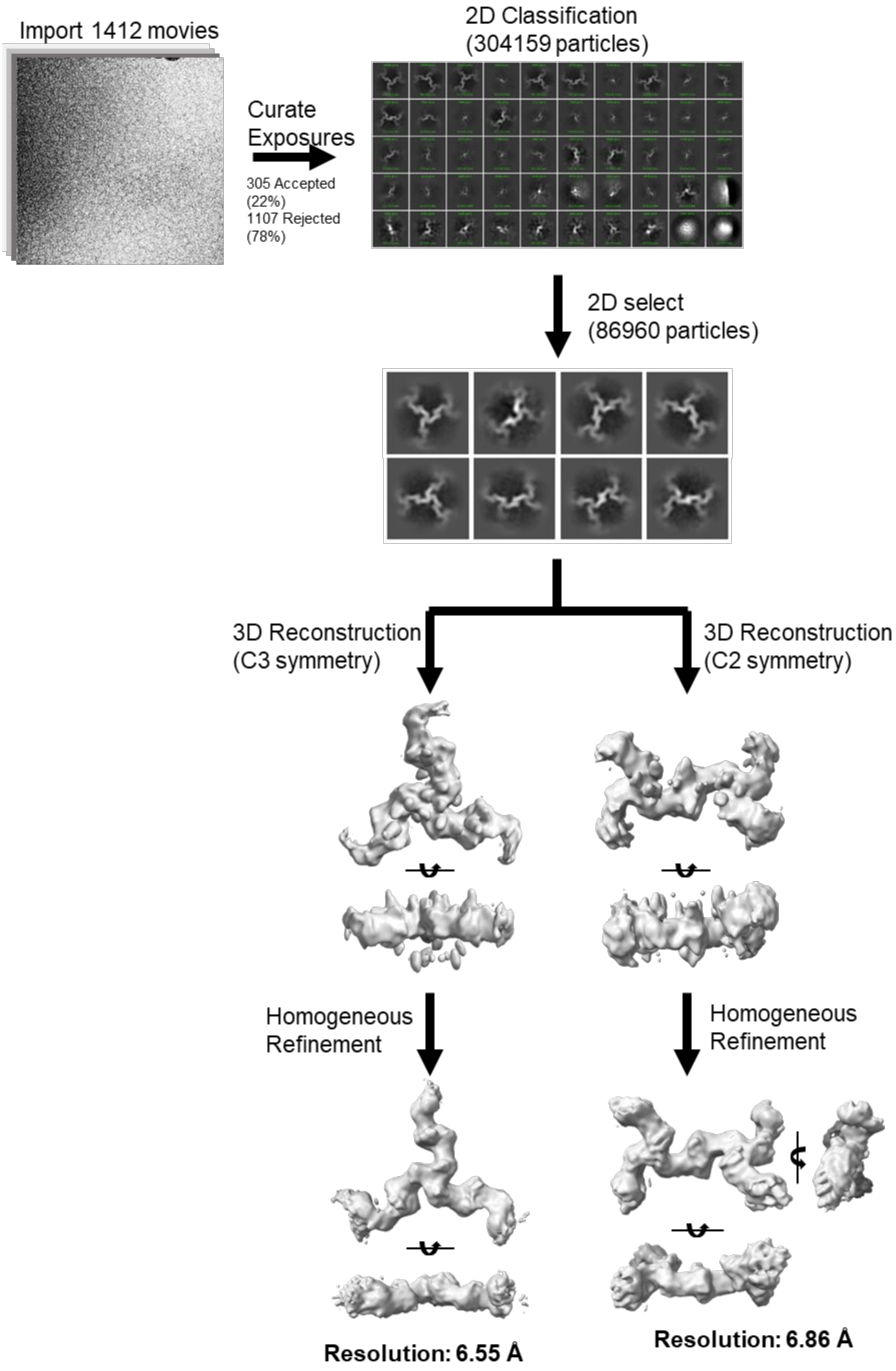

Supplementary Figure 3. Workflow of cryo-EM for BcCotE

A total of 1,412 movies were collected using a Glacios microscope equipped with a Falcon 4i detector. The images were sorted, and 1,107 movies in which CotE mesh images could not be observed were rejected. The particles were autopicked and visually examined using CryoSPARC. Fifty 2D-classification classes were generated by CryoSPARC, of which eight classes (86,960 particles) were selected for the next process. Ab initio reconstruction was performed using C3 and C2 symmetries for the selected particles, allowing for the creation of a 3D map. The created maps underwent a homogeneous refinement process and resulted in maps with resolutions of 6.55 and 6.86 Å, respectively.

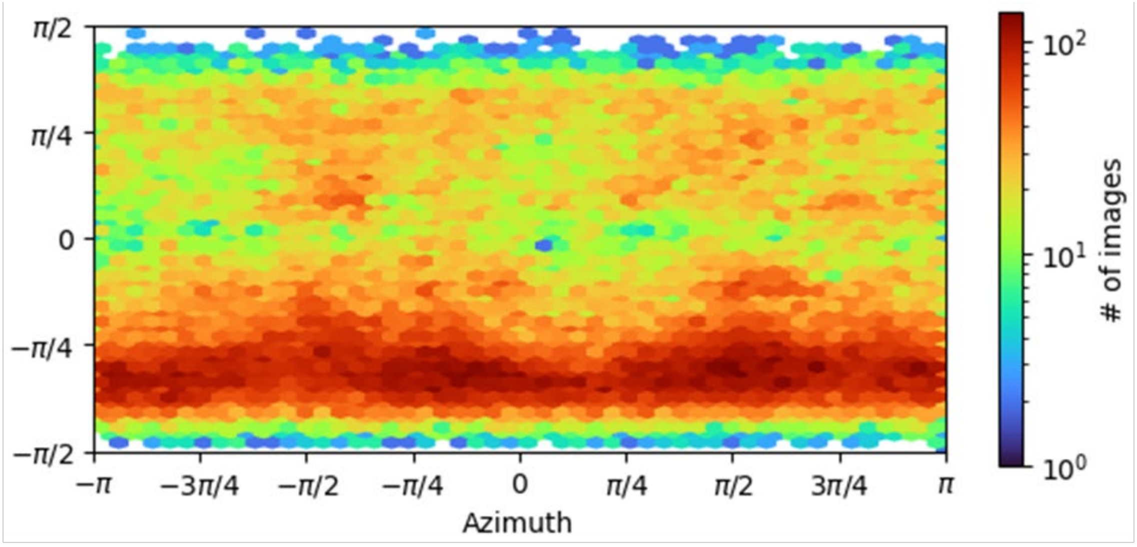

C3 symmetry orientation plot

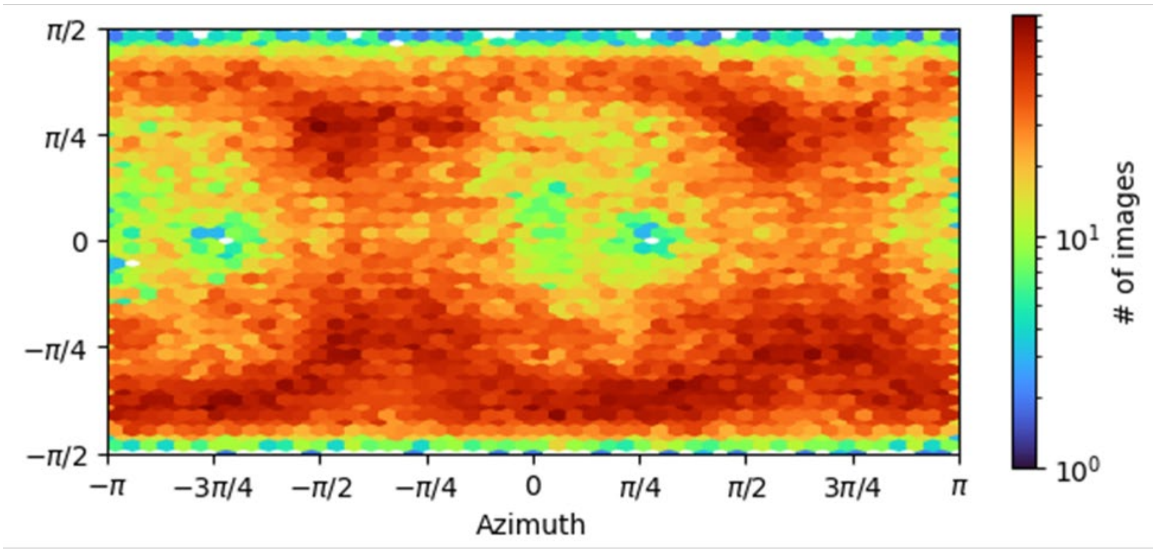

C2 symmetry orientation plot

Supplementary Figure 4. Orientation distribution plots for the C3 and C2 symmetry 3D reconstruction EM maps

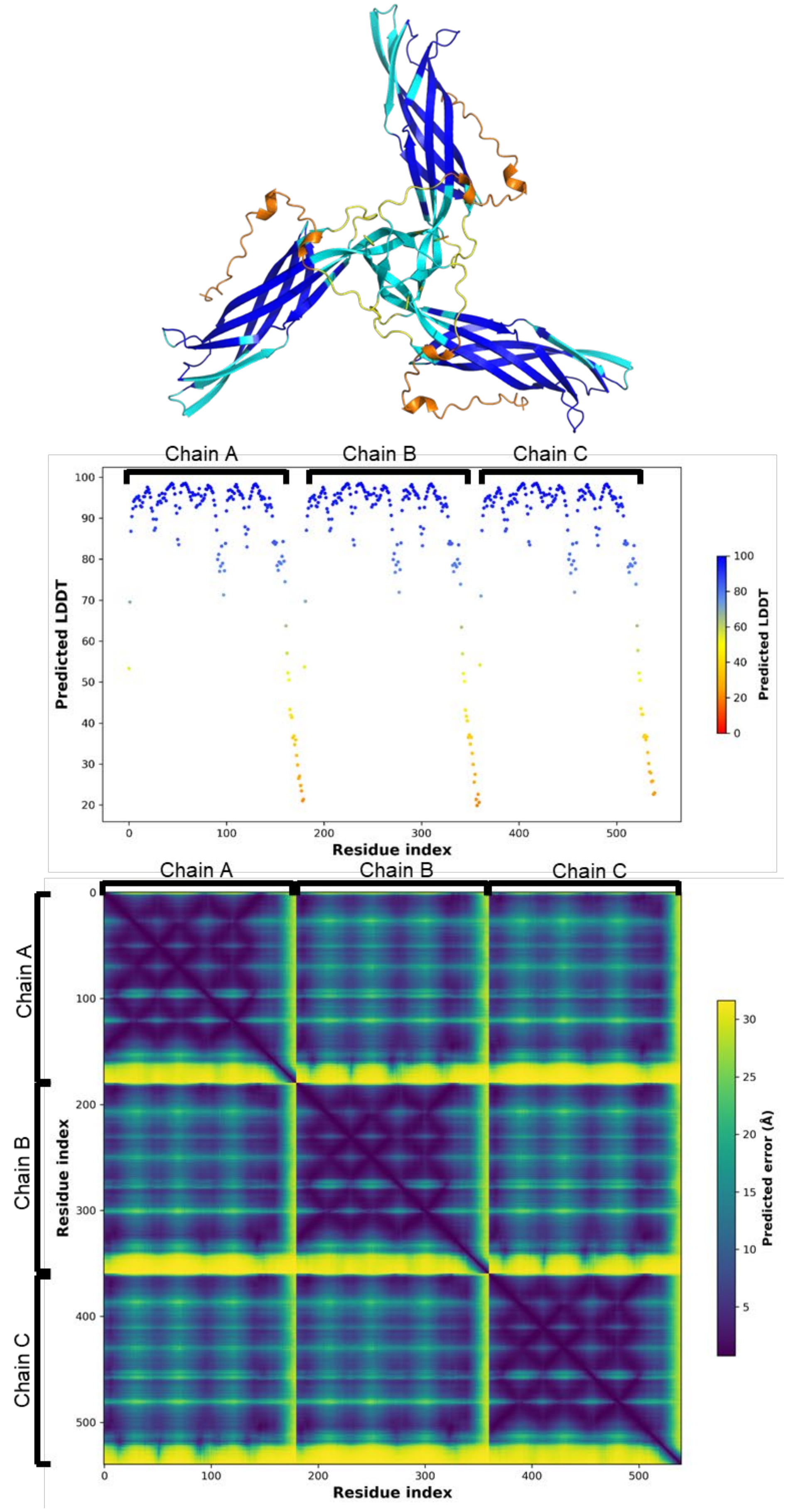

Chain A
Chain B
Chain C
Predicted LDDT
Residue index
Predicted LDDT
Chain A
Chain B
Chain C
Chain A
Chain B
Chain C
Residue index
Residue index
Predicted error (Å)

Supplementary Figure 5. The pLDDT and PAE plots for the AlphaFold2 predicted trimer model of the BcCotE protein

The predicted structure of the CotE trimer is shown at the top, with pLDDT values colored for each residue from blue to orange (pLDDT>= 90 : Blue, 70 <= pLDDT < 90 : Cyan, 50 <= pLDDT < 70 : Yellow, pLDDT < 50 : Orange), and the pLDDT plot and PAE plot are presented below. The Each chain is labeled in the plot.

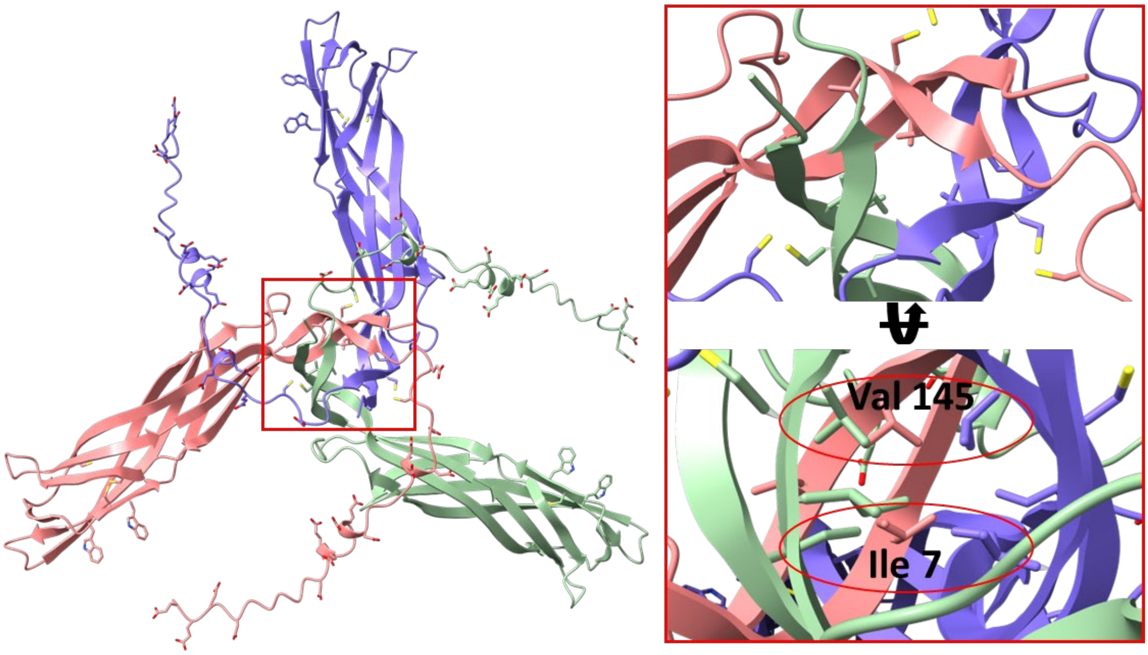

Supplementary Figure 6. The trimer model built by AlphaFold2 (left) and an enlarged image of the trimeric core region (right)

Enlarged images of the top and side views are shown on the right. The three Val145 and three Ile7 residues formed a hydrophobic core. Each CotE monomer is shown in green, red, or purple.

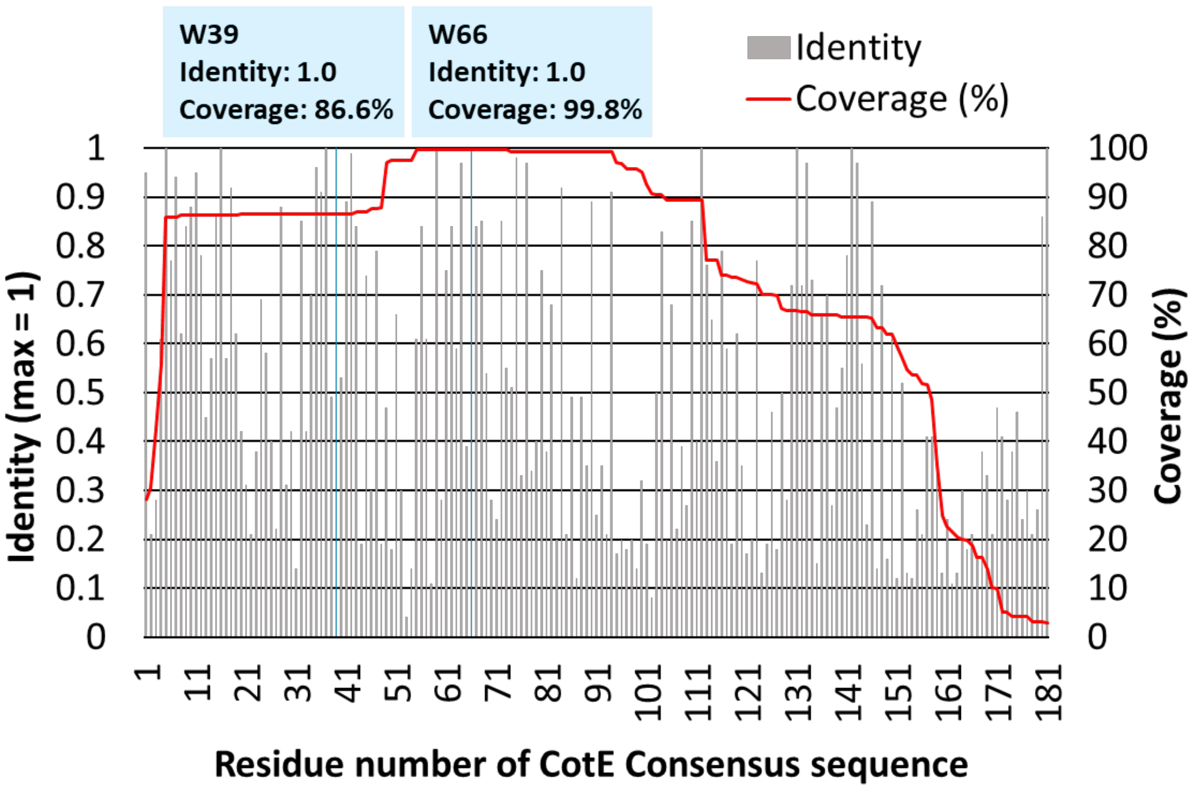

Supplementary Figure 7. Residue-wise identity and coverage ratios for the CotE consensus sequence derived from 464 BLASTp-aligned CotE sequences.

The amino acid sequence of *Bacillus cereus* CotE was analyzed using UniProt BLAST against the 'UniProtKB reference proteomes + Swiss-Prot' database with the following parameters: program, blastp; E-threshold, 10; matrix, BLOSUM62 (auto-selected); and hits, 1000. Filtering was disabled, and only the top-scoring HSP per hit was retained. After removing overlapping species, 464 non-redundant sequences were aligned to derive a consensus sequence.

The graph depicts identity (grey) and coverage (red) values for each residue within the consensus sequence, with significant deviations noted at specific regions. Residues corresponding to tryptophan positions 39 and 65 in the sequences are highlighted in cyan, and the specific values are indicated by cyan boxes above the cyan bars for each residue. Trp65 in *Bacillus cereus* CotE was re-positioned as Trp66 in the consensus alignment. For detailed sequence alignment and residue information across species, refer to 'Supplementary file 1.pdf'.

```
MSEFREIITK AVVGKGRKYT KSTHTCESNN
EPTSILGCWV INHSYEARKN GKHVEIEGFY
DVNTWYSFDG NTKTEVVTER VNYTDEVSIG
YRDKNFSGDD LEIIARVIQP PNCLEALVSP
NGNKIVVTVE REFVTEVVGE TKICVSV/NPE
GCVESDDDFQ IADDEFEELD PNFIVDAEEE
```

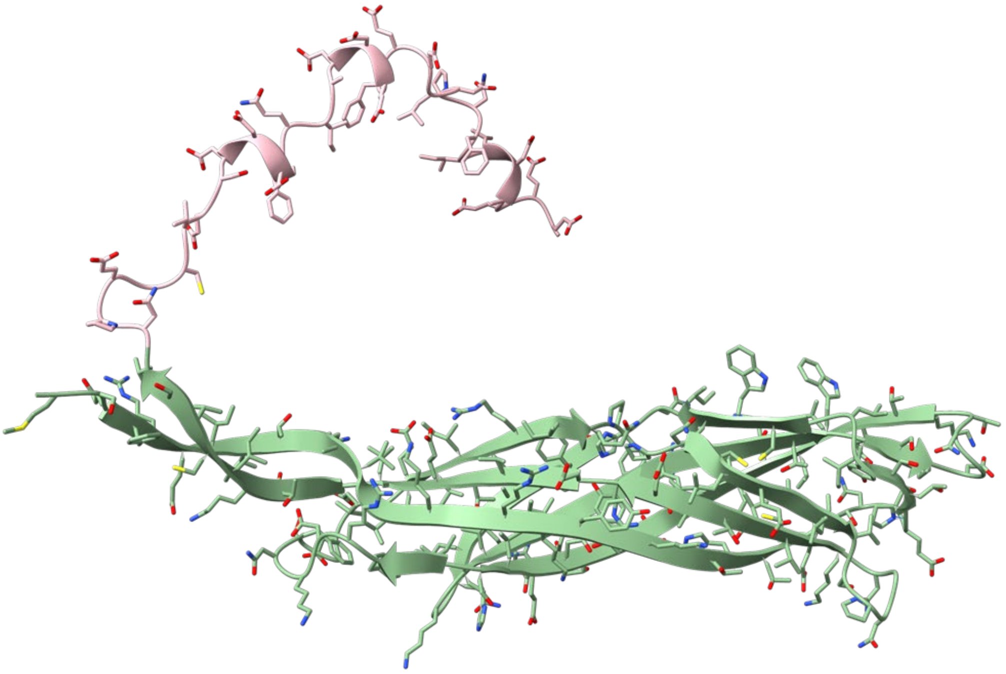

Supplementary Figure 8. Amino acid sequence of the BcCotE protein and model of the CotE monomer predicted by AlphaFold2

In the amino acid sequence, the N-terminal body and C-terminal tail domains are distinguished using the '/' mark. Glutamate and aspartate residues with negative charges in the C-terminal tail domain are highlighted in red. In the CotE monomer model, the N-terminal body and C-terminal tail domains are shown in green and pink, respectively.

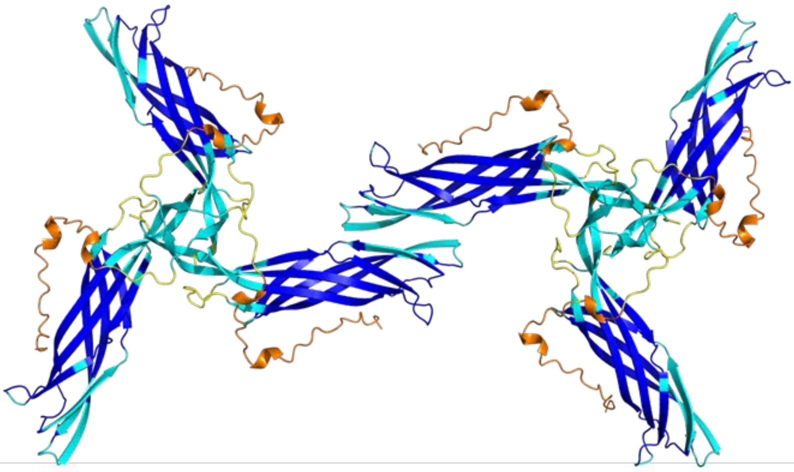

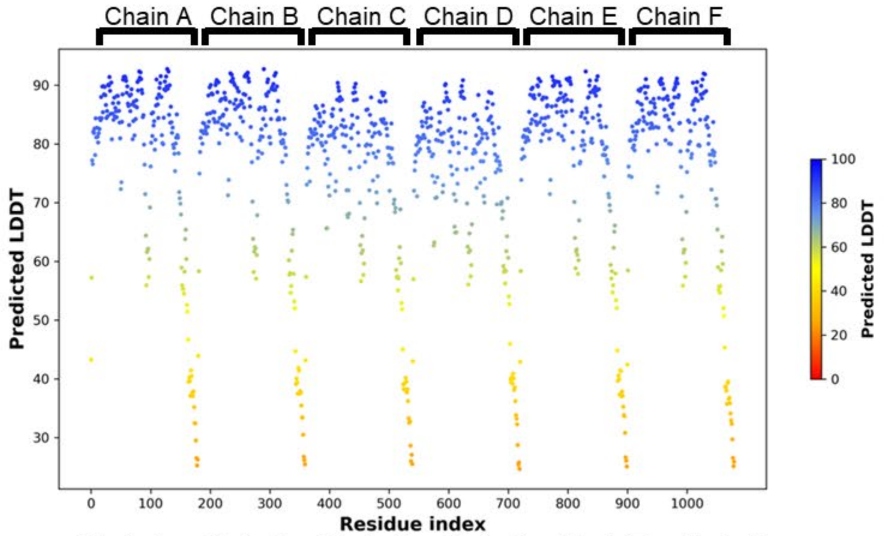
Chain A
Chain B
Chain C
Chain D
Chain E
Chain F
Predicted LDDT
90
80
70
60
50
40
30
0
100
200
300
400
500
600
700
800
900
1000
Residue index
100
80
60
40
20
0
Predicted LDDT

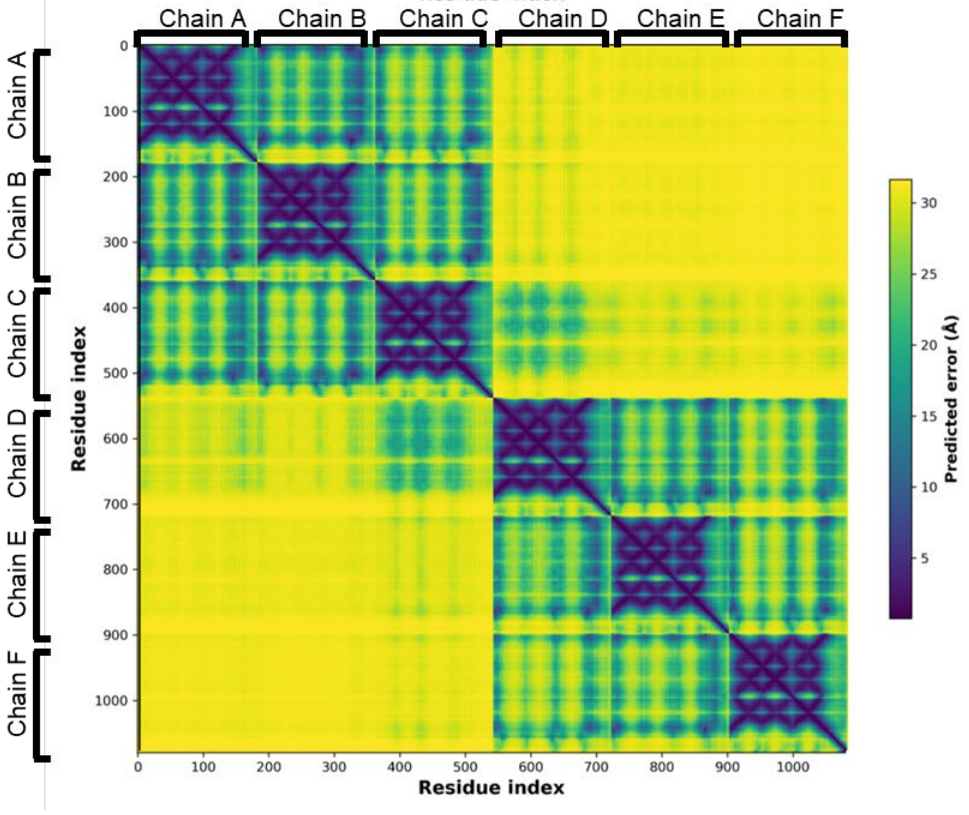
Chain A
Chain B
Chain C
Chain D
Chain E
Chain F
Chain A
Chain B
Chain C
Chain D
Chain E
Chain F
Residue index
0
100
200
300
400
500
600
700
800
900
1000
0
100
200
300
400
500
600
700
800
900
1000
Residue index
30
25
20
15
10
5
Predicted error (Å)

Supplementary Figure 9. The pLDDT and PAE plots for the AlphaFold2 predicted hexamer model of the BcCotE protein

The predicted structure of the CotE hexamer is shown at the top, with pLDDT values colored for each residue from blue to orange (pLDDT>= 90 : Blue, 70 <= pLDDT < 90 : Cyan, 50 <= pLDDT < 70 : Yellow, pLDDT < 50 : Orange), and the pLDDT plot and PAE plot are presented below. Each chain is labeled in the plot.

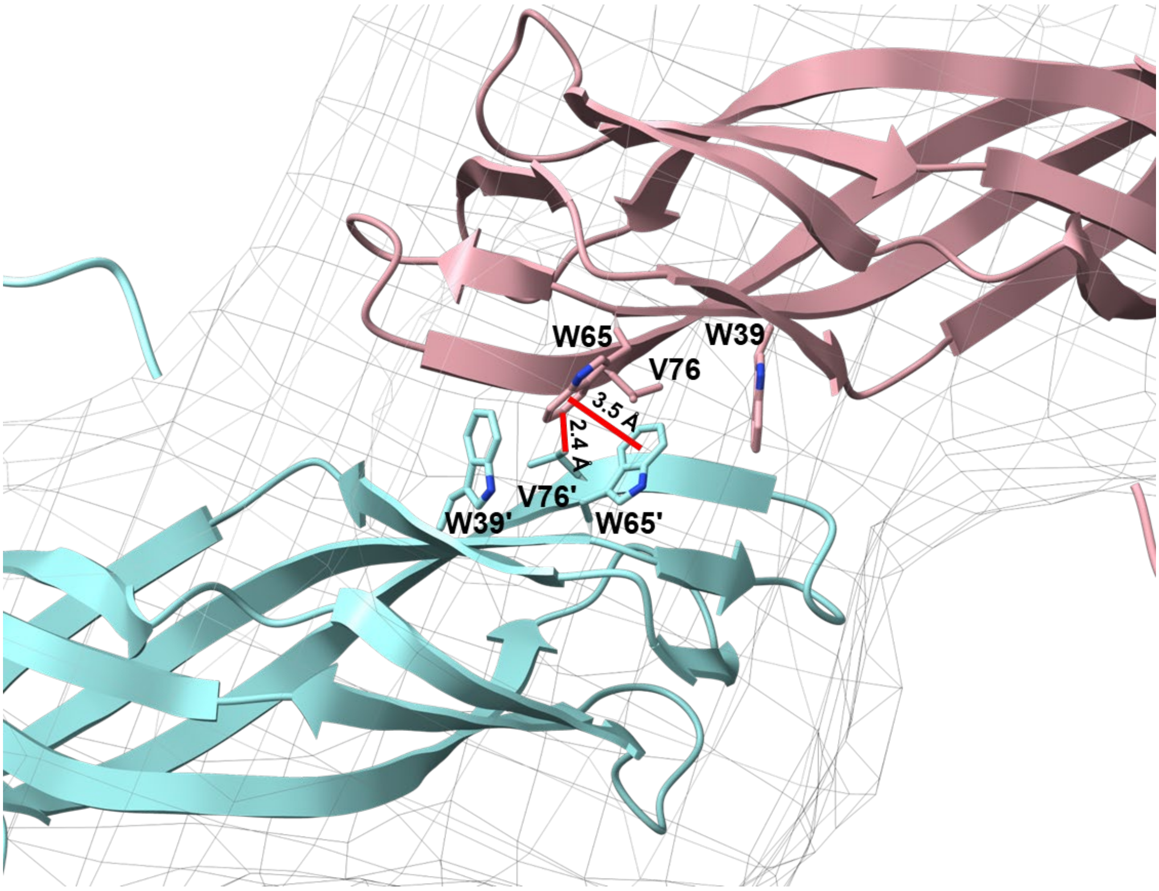

Supplementary Figure 10. Enlarged view of the dimeric interface in the AlphaFold model fitted to the determined EM map of the BcCotE protein

A magnified view of the dimeric interface between the two CotE trimers fitted to the C2 symmetry-refined map (Fig. 3d). Each trimer is colored light blue or pink, as shown in Fig. 3d. Protein structural models were aligned to the map using the *fitmap* function of ChimeraX and refined using Phenix. Residues W39, W65, and V76 of each protein contribute to the formation of a hydrophobic core-like structure.

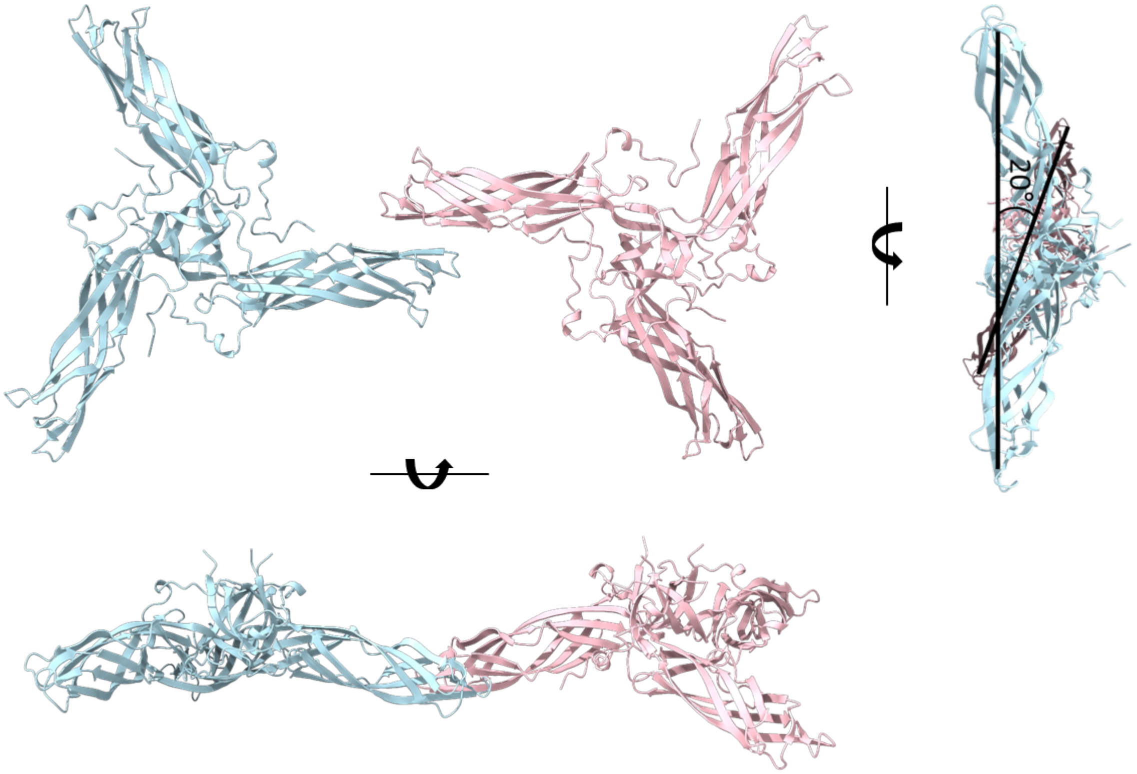

Supplementary Figure 11. Three orthogonal views of the predicted structure of the CotE hexamer by AlphaFold2 with a tilt angle of approximately 20°

The tilt angle of the AlphaFold2 hexamer prediction model. Each trimer is indicated in blue or pink. The tilt angle is confirmed to be approximately 20°.

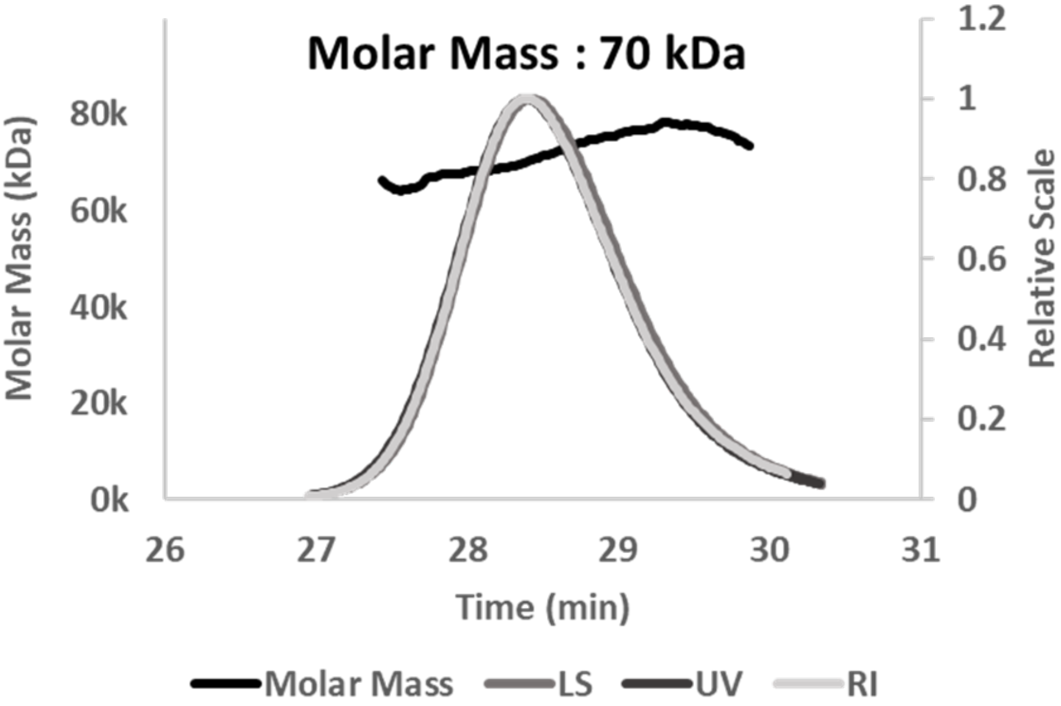

Supplementary Figure 12. Results of MALS analysis for the W65E mutation of BcCotE

The molar mass graph utilizes the left Y-axis scale, while the Light Scattering (LS), Ultraviolet (UV), and Refractive Index (RI) graphs use the right Y-axis scale. The W65E-mutant protein was found to have a size of 70 kDa, approximately equal to the size of a trimer.

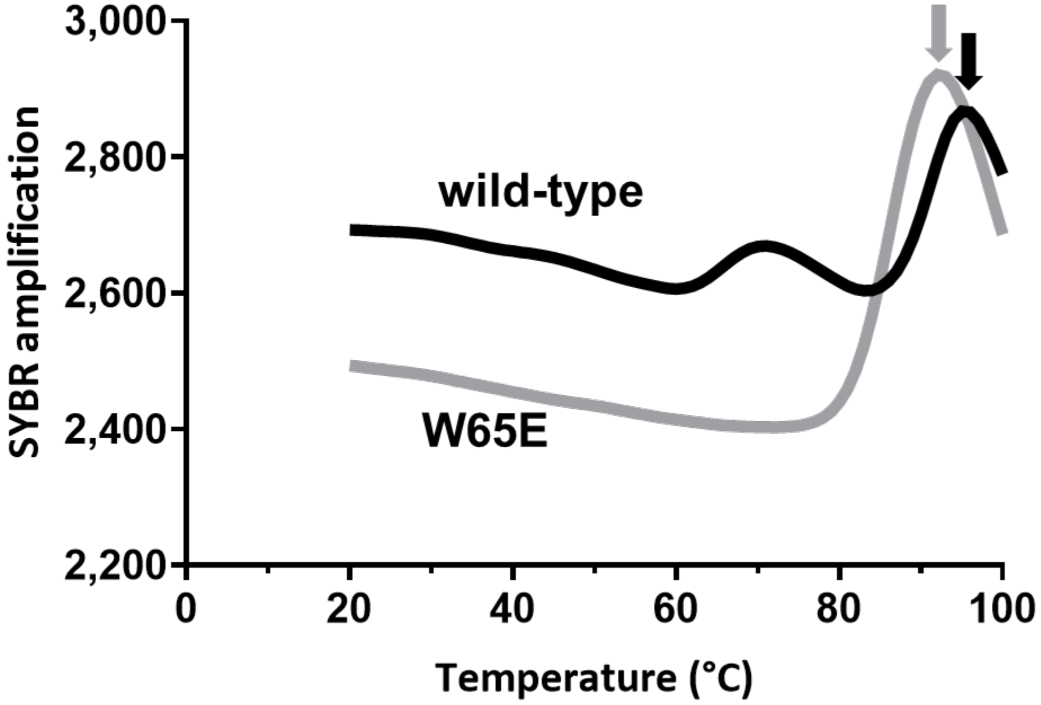

Supplementary Figure 13. Results of thermal shift assay for wild-type and W65E-mutant CotE protein

The melting temperatures (Tm) of the wild-type and W65E-mutant CotE proteins were determined by a thermal shift assay using the fluorescent dye SYBR Green. The major peaks (arrows) indicating the structural transition occur at approximately 90–95 °C for both proteins.

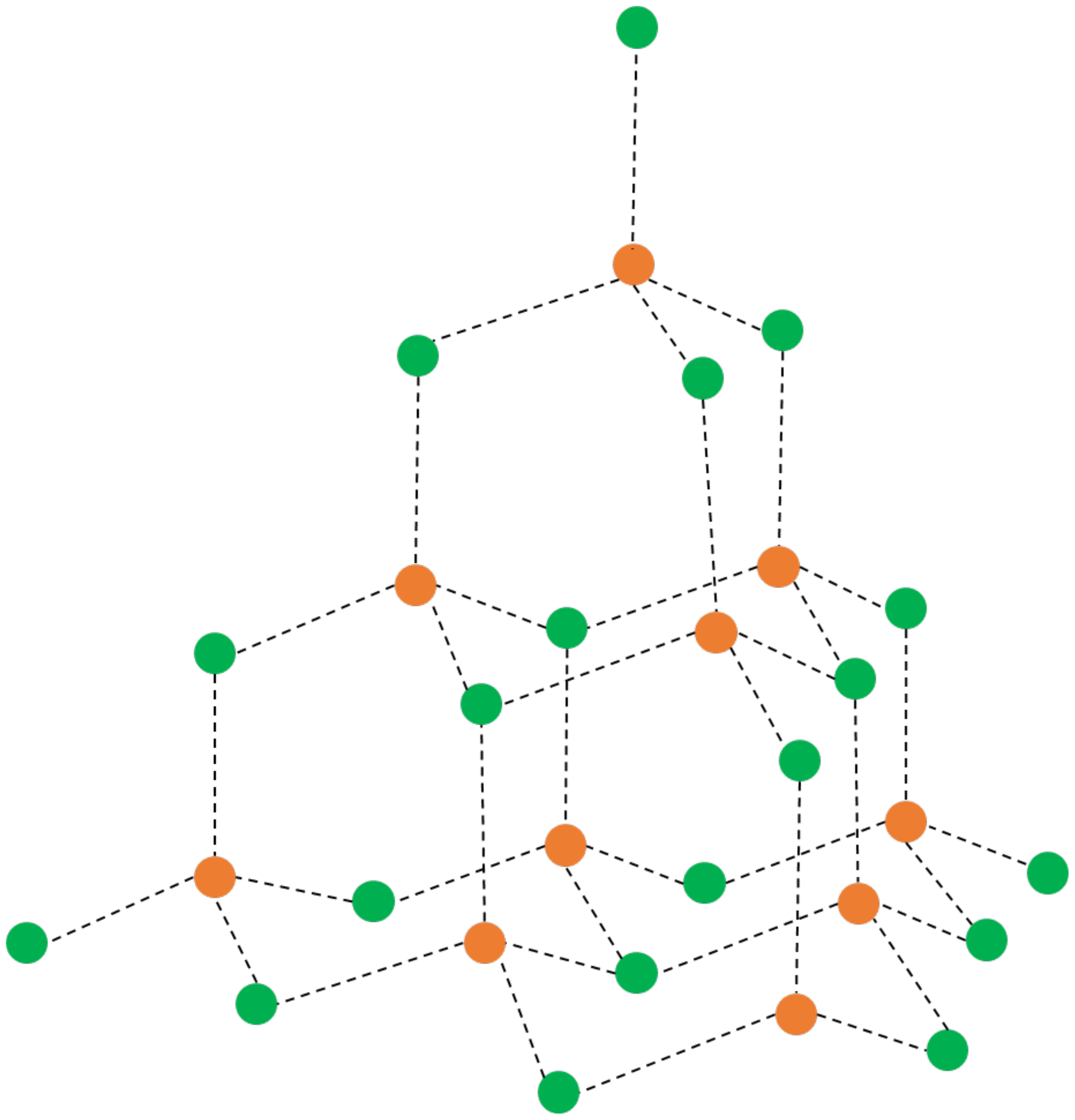

Supplementary Figure 14. Schematic diagram of the arrangement of carbon atoms in a diamond crystal

Each carbon atom (green and orange circles) is connected to four bonds, creating a structure similar to that of the model shown in Fig. 4.

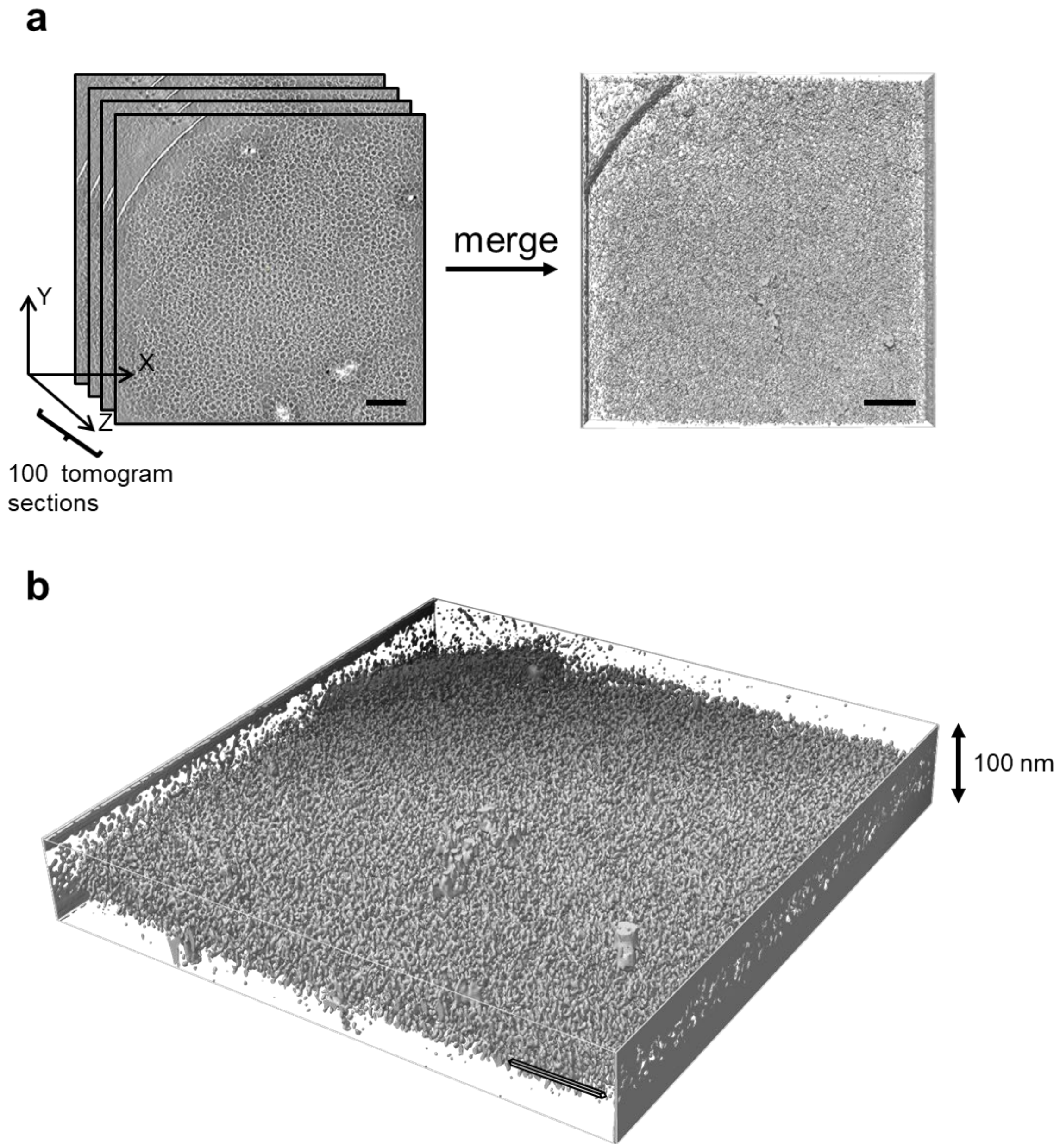

Supplementary Figure 15. Surface map observed in cryo-ET data of the CotE protein

a. The 100-nm thick CotE protein ET surface map was formed by merging 100 tomogram sections obtained by cryo-ET. Each section of the tomogram can be observed in Suppl. Movie 1. The black scale bar represents 100 nm.

b. A bird's-eye view of the CotE ET surface map is shown in (a). The z-axis thickness of the entire structure is 100 nm, but the region where the CotE protein structure is observed ranges from approximately 30–70 nm. The black scale bar represents 100 nm.

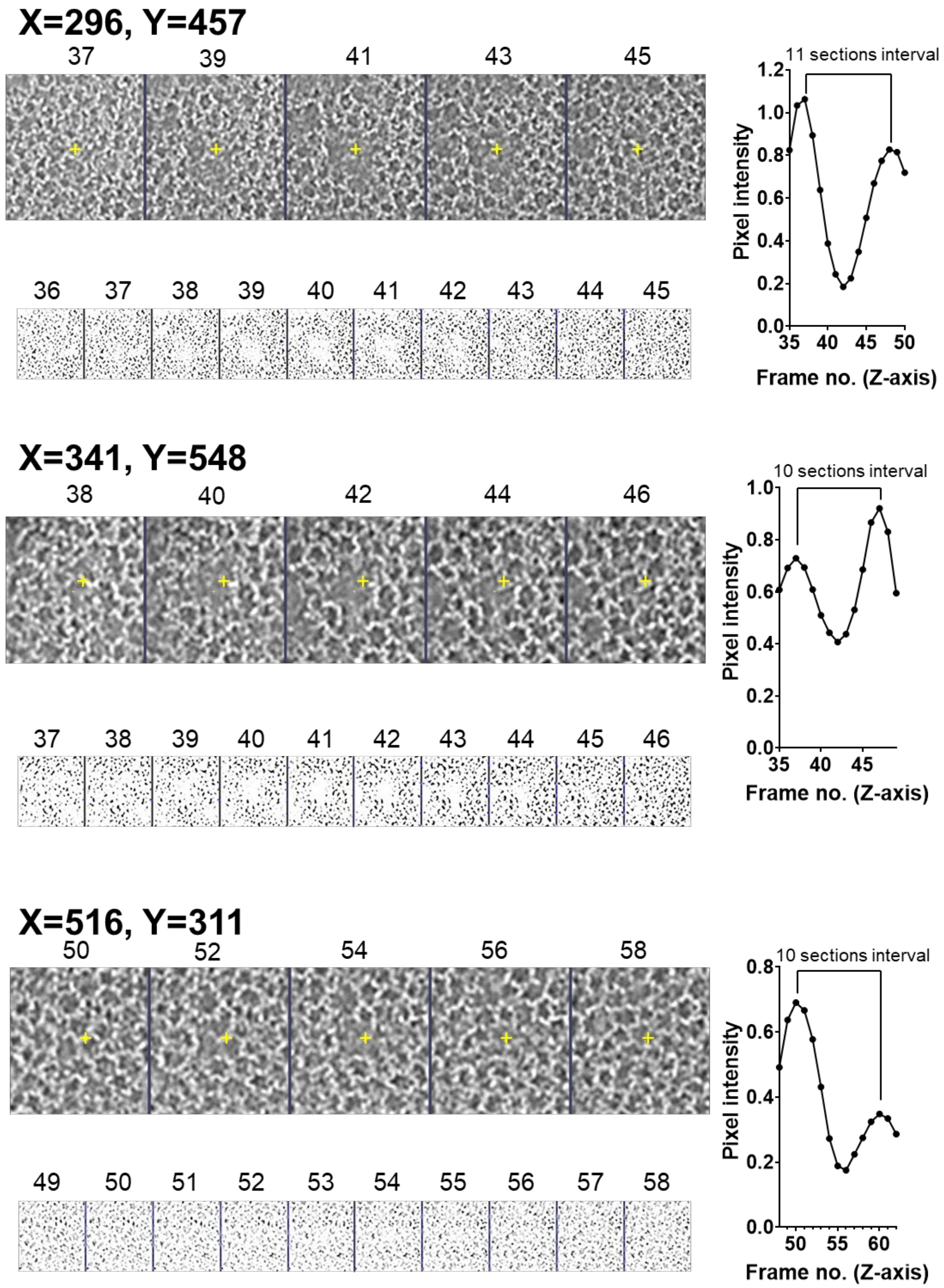

Supplementary Figure 16. Observation of CotE protein showing changes in mesh structure at intervals of approximately 10 nm

Representative images illustrate changes in the CotE protein mesh structure as the Z-axis frame

number of the tomogram increases, as depicted in Fig. 5b. The coordinates of the center (yellow cross) of each image are displayed above each data point. The mesh structure at the center of the tomogram section becomes invisible and then reappears at approximately 10 frames, as shown at a two-frame interval. Below the processed image of 10 consecutive tomogram sections, the empty space expressed in white at the center can be observed to grow and then shrink. The graph on the right tracks the pixel intensity at the coordinates indicated by the yellow crosses on the left.

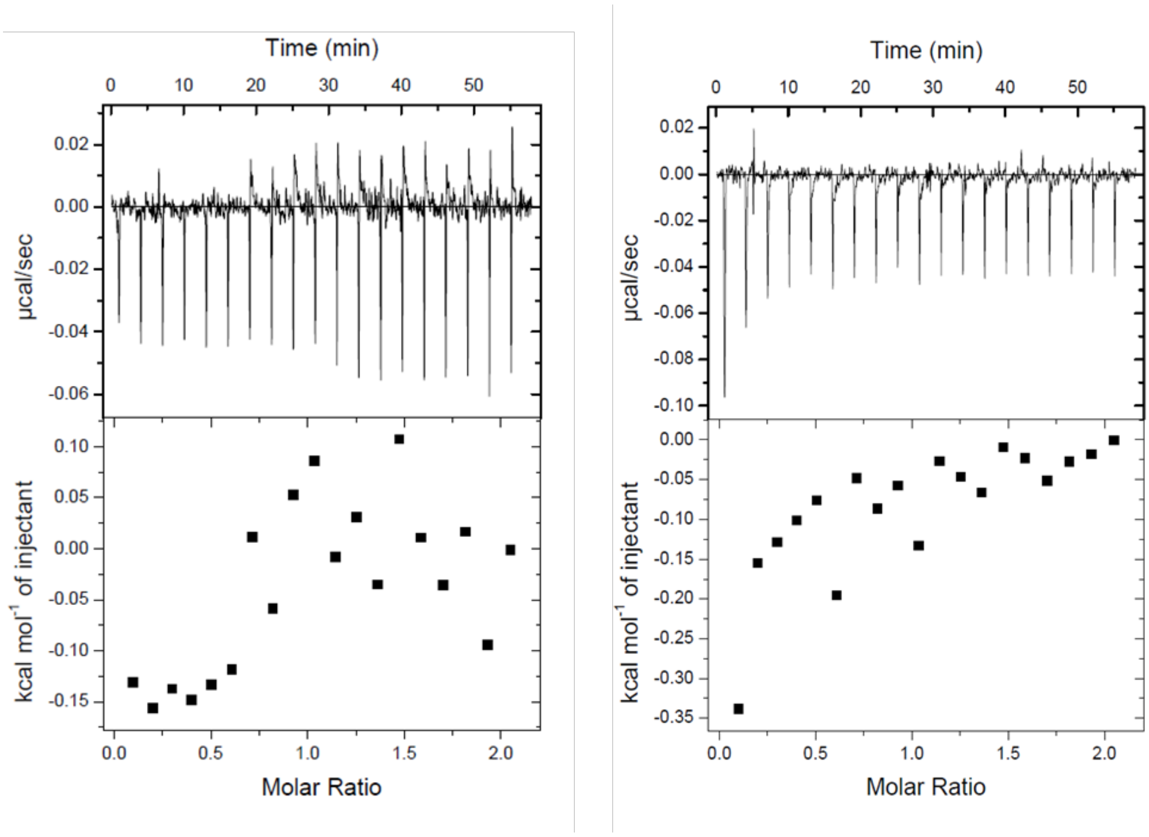

Supplementary Figure 17. Calorimetric titration profiles and fitting plots of wild-type CotE by isothermal titration calorimetry (ITC)

The wild-type CotE proteins (100 µM) in PBS were placed in the sample cell, while 1 mM $CaCl_2$ (left) or 1 mM DPA (right) was used as the titrant in the syringe.

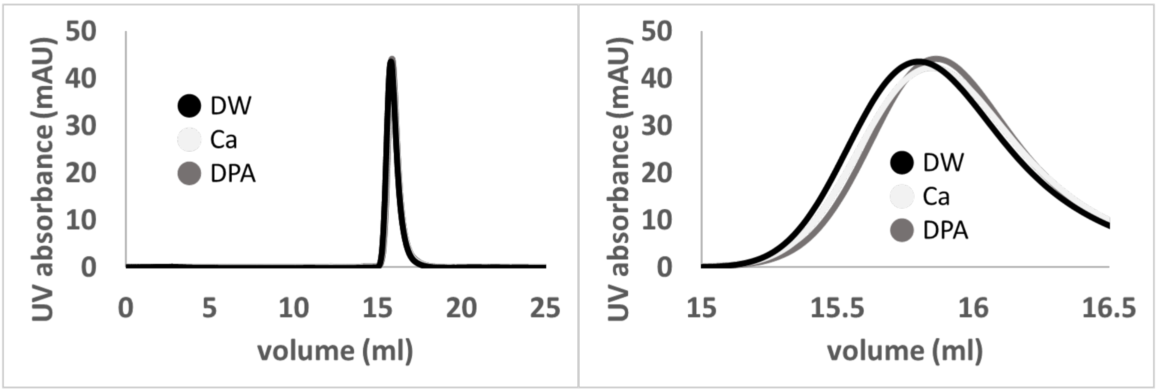

Supplementary Figure 18. SEC result plot of W65E-mutant CotE protein under three conditions

Size-exclusion chromatography (SEC) was performed using a Superdex 120 column for the W65E-mutant CotE protein under three conditions: no addition, addition of 5 mM Ca^{2+}, and addition of 5 mM DPA. The graph on the right presents an enlarged view of the peak regions from the left graph. The buffer contained 20 mM Tris-HCl (pH 8.0) and 150 mM NaCl. The black line represents the condition with no addition (DW denotes deionized water), the light gray line represents the addition of Ca^{2+}, and the dark gray line represents the addition of DPA.

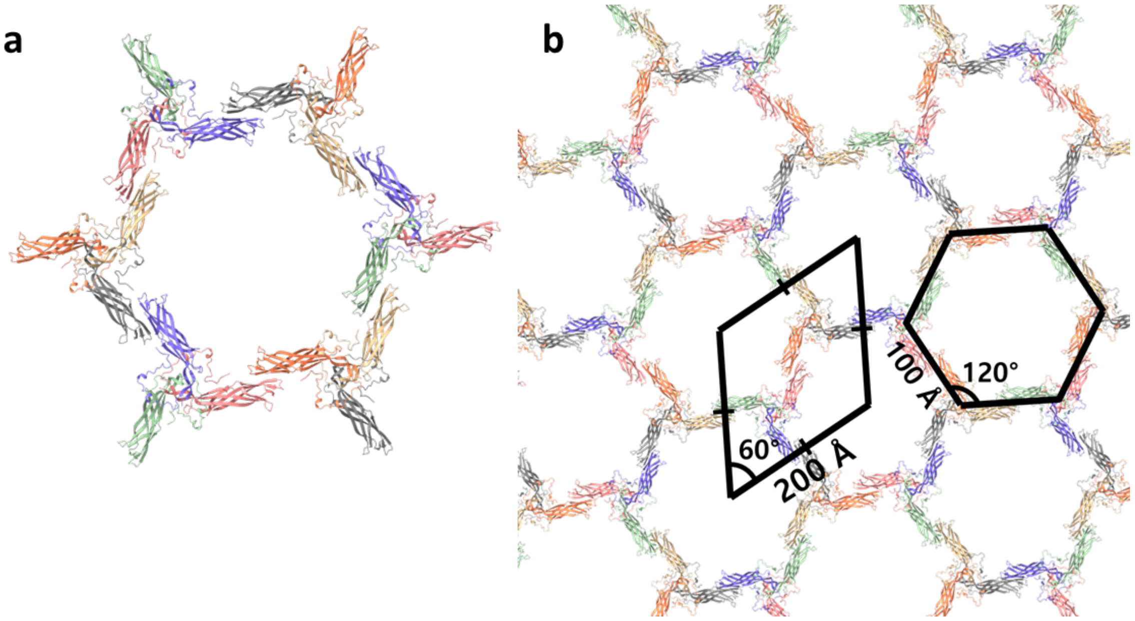

Supplementary Figure 19. Flattened planar array structural model of the CotE protein

a. An 18-mer CotE hexagonal ring model based on the AlphaFold2 hexamer predicted the structure. Since the predicted hexamer structure is not perfectly flat, the angle between the two trimers was adjusted to form a hexagon in one plane.

b. CotE honeycomb network macrocomplex model created by connecting the hexagonal ring structures shown in (a). The distance between each hexagon is approximately 200 Å, and the length of one side of each hexagon is approximately 100 Å.

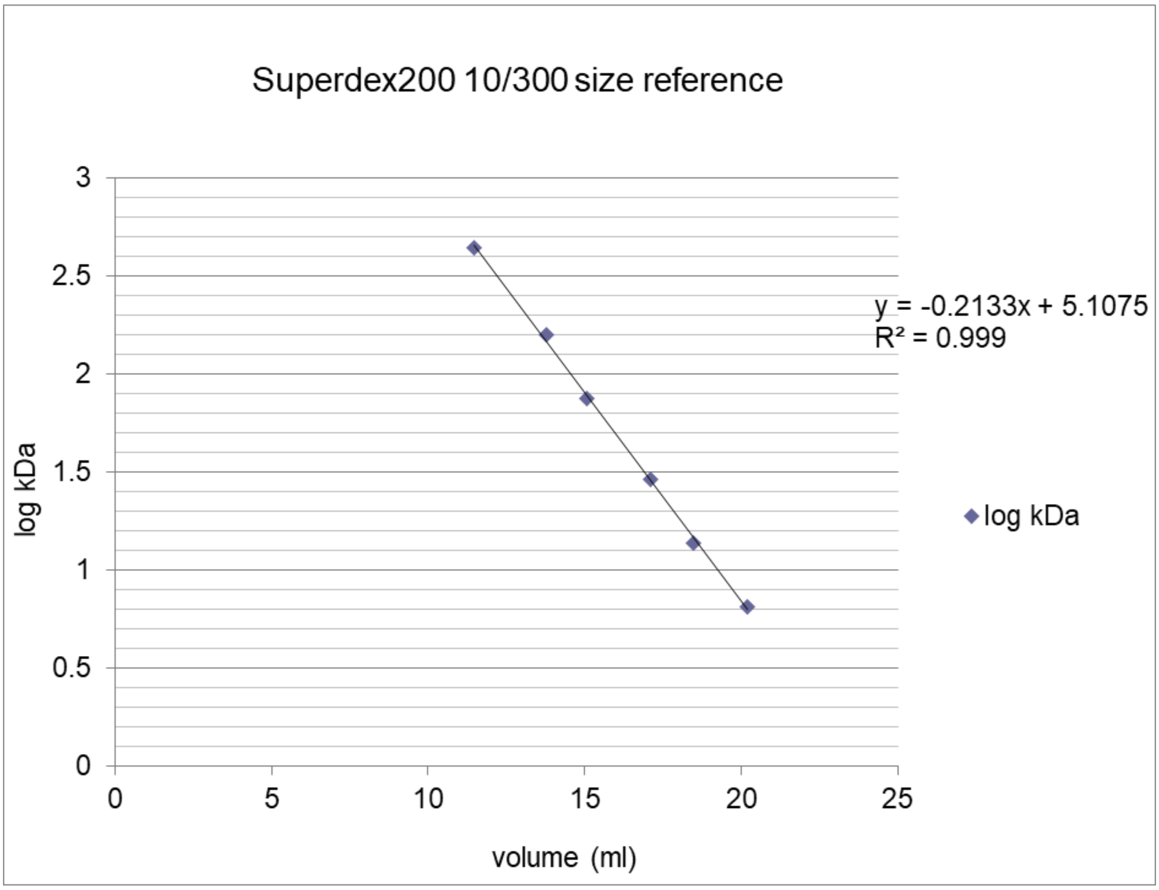

Supplementary Figure 20. Calibration curve of a Superdex 200 Increase 10/300 GL size-exclusion chromatography column

A calibration curve was obtained by plotting the elution volumes of a series of reference proteins against their known molecular weights (log kDa) using a Superdex 200 Increase 10/300 GL size-exclusion chromatography column. Reference proteins were obtained using Gel Filtration Calibration Kits (LMW and HMW) from Cytiva.

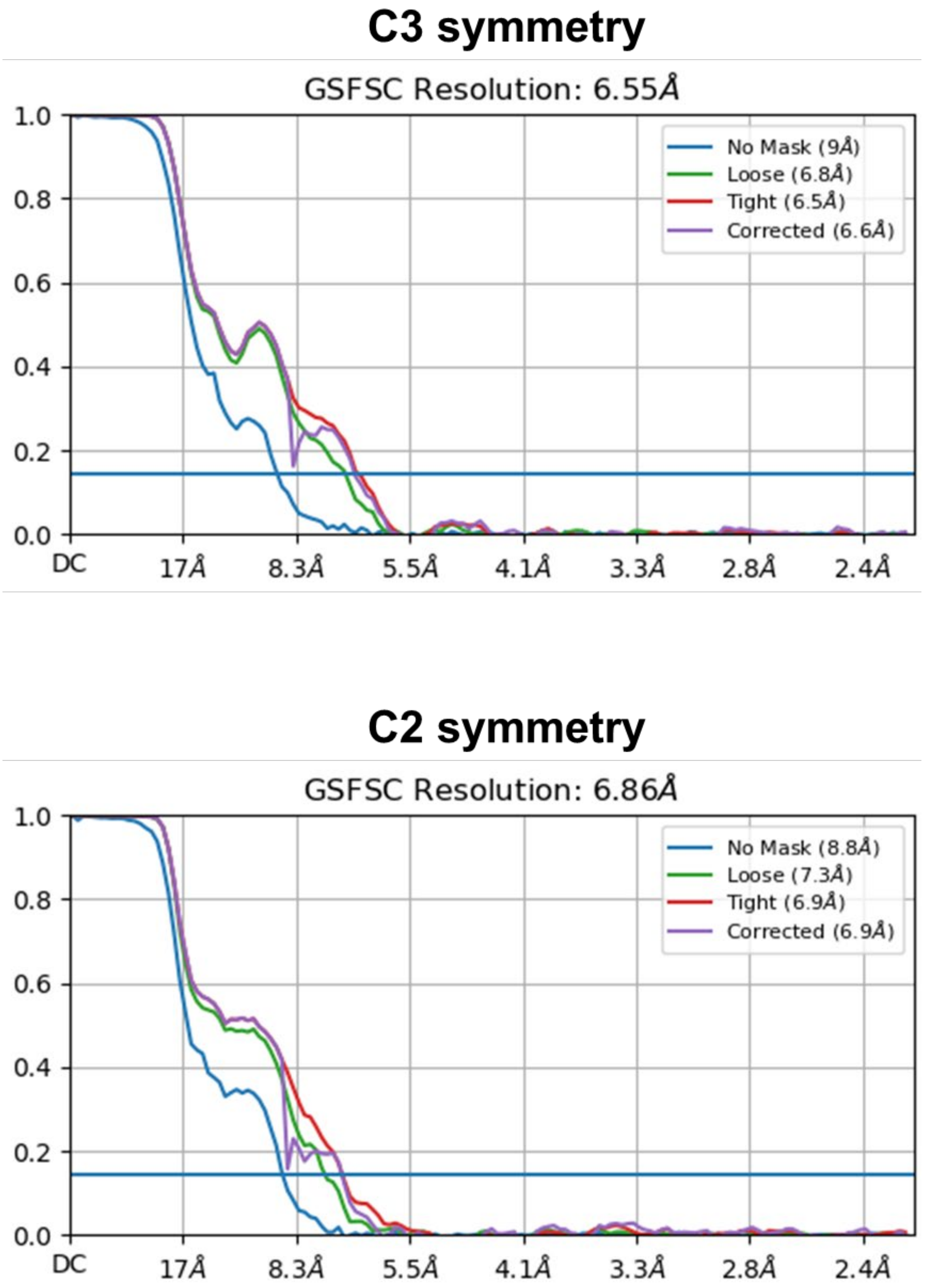

Supplementary Figure 21. Gold-standard Fourier Shell Correlation (GSFSC) resolution graphs for CotE 3D reconstruction electron microscopy (EM) maps

GSFSC resolutions when creating 3D reconstruction EM maps, as shown in Fig. 2c.

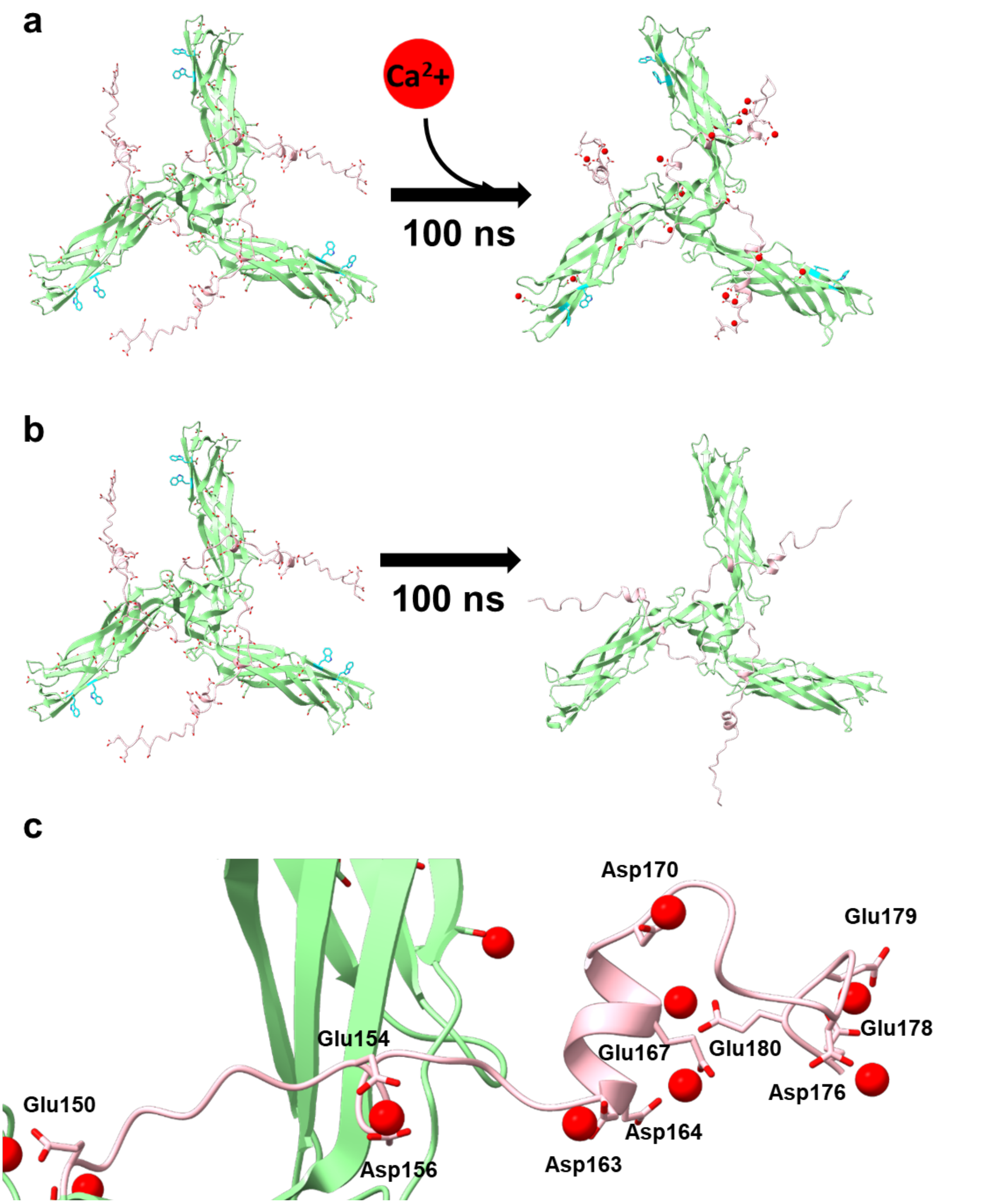

Supplementary Figure 22. Comparison of the shape of the C-terminal loop after molecular dynamics (MD) simulation of the CotE trimer for 100 ns

a. The 100-ns MD simulation of the wild-type CotE protein in the presence of 5 mM Ca^{2+}, as shown in Fig. 6c.

b. The 100-ns MD simulation of the wild-type CotE protein in the absence of Ca^{2+}.

c. Enlarged view of the C-terminal loop from the 100 ns MD simulation in the presence of 5 mM Ca^{2+}, as shown in Fig. 6c. Eleven negatively charged residues in the C-terminal loop (pink) interact with Ca^{2+} (red). The residues that interact with Ca^{2+} are Glu150, Glu154, Asp156, Asp163, Asp164, Glu167, Asp176, Glu179, and Glu180.

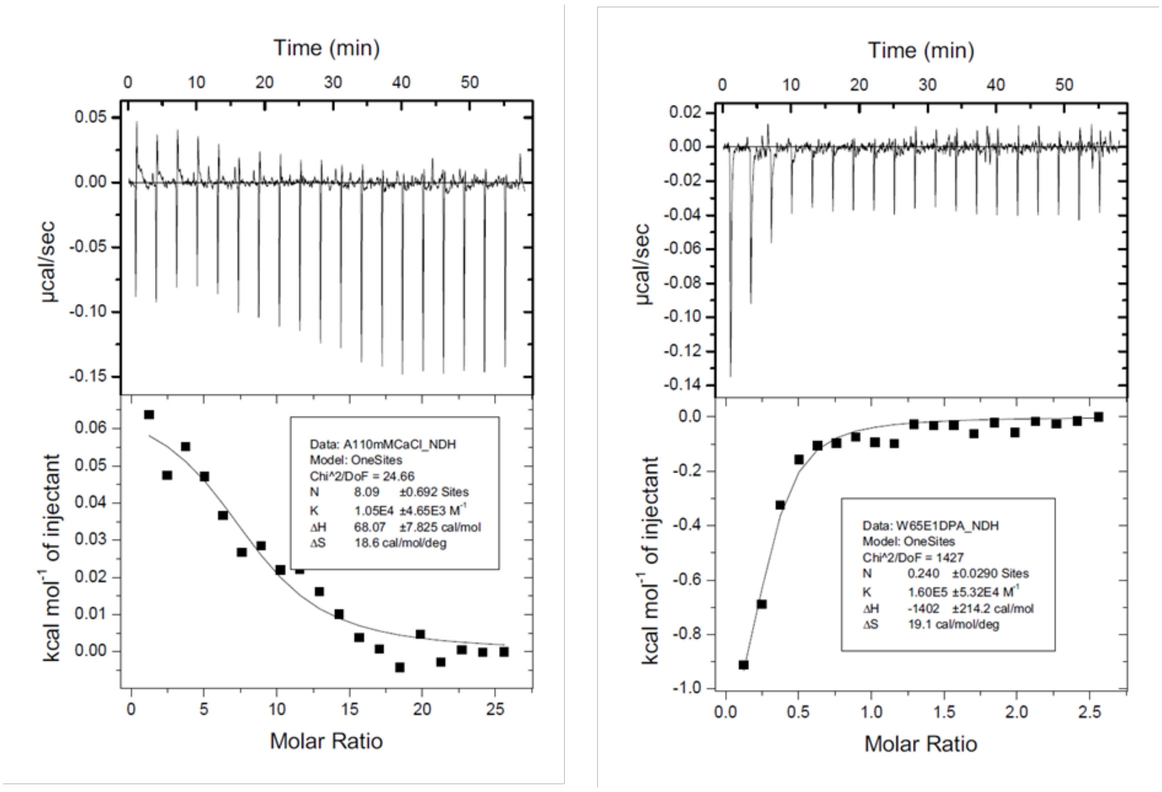

Supplementary Figure 23. Calorimetric titration profiles and fitting plots of W65E-mutant CotE by isothermal titration calorimetry (ITC)

The W65E-mutant CotE proteins (100 μM) in PBS were placed in the sample cell, while 10 mM $CaCl_2$ (left) or 1 mM DPA (right) was used as the titrant in the syringe.

Gene	Vector	Primer orientation	Enzyme site	Sequence
Wild-type CotE	**pET28a**	Forward	NcoI	5'- GCGCCATGGCGTCCGAATTTAGAGAG-3'
		Reverse	XhoI	5'- GCGCTCGAGTTACTCTTCTTCTGCATC-3'
W65E mutant CotE	**pET28a**	Forward	Point mutation	5'- CGTTAACACCGAGTACTCTTTCG-3'
		Reverse	Point mutation	5'- CGAAAGAGTACTCGGTGTTAACG-3'
C-terminal loop deletion mutant (ΔCT) CotE	**pET28a**	Forward	NcoI	5'- GCGCCATGGCGTCCGAATTTAGAGAG--3'
		Reverse	XhoI	5'-GCGCTCGAGTTAAGATTCAACGC-3'

Supplementary Table 1. Primers designed for wild-type, W65E-mutant, and ΔCT-mutant CotE

	C3 symmetry of CotE	C2 symmetry of CotE
Magnification	92000	92000
Voltage (kV)	200	200
Camera	Falcon 4 (Glacios)	Falcon 4 (Glacios)
Frame exposure time (s)	14.77	14.77
Exposure fractionations (n)	40	40
Electron exposure ($e^-/Å^2$)	50	50
Defocus range (μm)	-2.0 to -1.0	-2.0 to -1.0
Pixel size (Å)	1.1	1.1
Symmetry imposed	C3	C2
Box size (pixel)	300	300
Micrographs collected (n)	1412	1412
Segments extracted (n)	304159	304159
Segments after Class2D (n)	86960	86960
Map resolution (Å)	6.55	6.86
FSC threshold	0.143	0.143

Supplementary Table 2. Cryo-EM data collection and processing statistics

Supplementary Movie 1 (separate file). Visualization of representative BcCotE tomograms

Supplementary file 1 (separate file). 464 BLASTp-aligned CotE sequences and their consensus sequence
